# Life and Death of Selfish Genes: Comparative Genomics Reveals the Dynamic Evolution of Cytoplasmic Incompatibility

**DOI:** 10.1093/molbev/msaa209

**Published:** 2020-08-14

**Authors:** Julien Martinez, Lisa Klasson, John J Welch, Francis M Jiggins

**Affiliations:** 1 Department of Genetics, University of Cambridge, Cambridge, United Kingdom; 2 MRC-University of Glasgow Centre for Virus Research, University of Glasgow, Glasgow, United Kingdom; 3 Molecular Evolution, Department of Cell and Molecular Biology, Uppsala University, Uppsala, Sweden

**Keywords:** *Wolbachia*, cytoplasmic incompatibility, *cif* genes evolution

## Abstract

Cytoplasmic incompatibility is a selfish reproductive manipulation induced by the endosymbiont *Wolbachia* in arthropods. In males *Wolbachia* modifies sperm, leading to embryonic mortality in crosses with *Wolbachia*-free females. In females, *Wolbachia* rescues the cross and allows development to proceed normally. This provides a reproductive advantage to infected females, allowing the maternally transmitted symbiont to spread rapidly through host populations. We identified homologs of the genes underlying this phenotype, *cifA* and *cifB*, in 52 of 71 new and published *Wolbachia* genome sequences. They are strongly associated with cytoplasmic incompatibility. There are up to seven copies of the genes in each genome, and phylogenetic analysis shows that *Wolbachia* frequently acquires new copies due to pervasive horizontal transfer between strains. In many cases, the genes have subsequently acquired loss-of-function mutations to become pseudogenes. As predicted by theory, this tends to occur first in *cifB*, whose sole function is to modify sperm, and then in *cifA*, which is required to rescue the cross in females. Although *cif* genes recombine, recombination is largely restricted to closely related homologs. This is predicted under a model of coevolution between sperm modification and embryonic rescue, where recombination between distantly related pairs of genes would create a self-incompatible strain. Together, these patterns of gene gain, loss, and recombination support evolutionary models of cytoplasmic incompatibility.

## Introduction

Maternally transmitted bacteria in the genus *Wolbachia* commonly manipulate the reproduction of their arthropod hosts by inducing cytoplasmic incompatibility (CI). In the simplest case, CI causes embryonic mortality in crosses between symbiont-infected males and uninfected females (unidirectional CI). Because *Wolbachia-*infected females can still reproduce successfully with infected males, this provides them with a fitness advantage. Above a certain threshold in *Wolbachia* frequency, CI allows the infection to rapidly spread in the host population, even if it induces moderate fitness costs (Turelli et al. 1992). This is thought to have contributed to the remarkable evolutionary success of *Wolbachia*, which is estimated to infect around half of terrestrial arthropod species (Weinert et al. 2015).

Cytological studies have revealed that CI results from cytogenetic defects affecting the paternal chromosomes in early embryogenesis (Callaini et al. 1997; Tram and Sullivan 2002). As *Wolbachia* is not present in mature sperm, this suggests that *Wolbachia* modifies the sperm of infected males during spermatogenesis (the modification function). This leads to development failing unless the zygote inherits a *Wolbachia* from the mother that is able to rescue embryonic development (the rescue function) (Callaini et al. 1997; Tram and Sullivan 2002). When the male and female parents are infected with different *Wolbachia* strains, it is also common to find that the cross is incompatible (bidirectional CI) (O’Neill and Karr 1990; Bordenstein et al. 2001; Atyame et al. 2014). This suggests the modification and rescue factors must match each other for development to proceed normally (Poinsot et al. 2003).

Recent work on the *Wolbachia* strains *w*Mel and *w*Pip from *Drosophila melanogaster* and *Culex pipiens* has found that the bacterial genes *cifA* and *cifB* are sufficient to induce CI (Beckmann et al. 2017; Lepage et al. 2017; Chen et al. 2019; Shropshire and Bordenstein 2019). In both strains *cifA* is located directly upstream of *cifB*, and it is thought that *cifA* and *cifB* are transcribed as a single operon (Beckmann et al. 2017) (although this has been questioned by Shropshire and Bordenstein [2019]). In both *Wolbachia* genomes the genes are found within a prophage called WO, and the proteins encoded by the two genes bind each other (Beckmann et al. 2017; Chen et al. 2019).

In *Drosophila*, expressing *cifA^wMel^* in the female germline rescues CI in crosses with *w*Mel*-*infected males (Shropshire et al. 2018; Shropshire and Bordenstein 2019). Unexpectedly, in transgenic flies the modification of sperm requires both *cifA^wMel^* and *cifB^wMel^* to be expressed in the male germline (Lepage et al. 2017; Shropshire and Bordenstein 2019; Shropshire et al. 2020). This suggests that both genes are required for the modification function but only one gene for rescue and has been referred to as the “two-by-one” model of CI by some authors (Shropshire and Bordenstein, 2019). One hypothesis to explain this is that *cifA* and *cifB* together modify sperm in a way that is lethal unless reversed by *cifA* in the early embryo. Other authors have proposed that *cifB* may be the only toxin in sperm and that *cifA* could act as an antitoxin both in embryos and in sperm (Beckmann, Bonneau, et al. 2019). In this model, the requirement of *cifA* in sperm would be due to the fact that it protects maturing sperm cells from the toxic effect of *cifB* (Beckmann, Bonneau, et al. 2019). This model requires further validation as experiments have so far failed to detect *cifB* being transferred to females on mating (Beckmann and Fallon 2013).

There appear to be at least two distinct molecular mechanisms by which these genes cause CI, which are illustrated by two paralogous pairs of *cifA–cifB* genes in *w*Pip. In one case *cifB* has two PD-(D/E)XK domains, which encode DNase activity and are required for the modification of sperm (Chen et al. 2019). The other *cifB* paralog in *w*Pip has two PD-(D/E)XK domains that lack the residues required for nuclease activity (Chen et al. 2019). Instead, a deubiquitylating domain, which cleaves ubiquitin from proteins, is required for the sperm modification function (Beckmann et al. 2017; Beckmann, Sharma, et al. 2019). Based on these distinct molecular functions, *cif* genes with DNase activity are also known as *cinA* and *cinB*, and *cif* genes with deubiquitinase activity are known as *cidA* and *cidB* (Beckmann, Bonneau, et al. 2019). Following Beckmann, Bonneau, et al. (2019), we use the *cif* terminology to refer to CI factors regardless of their mode of action.

Homologs of *cifA* and *cifB* have been discovered in other *Wolbachia* genomes, allowing us to address questions around their evolution (Lepage et al. 2017; Lindsey et al. 2018). The *cifA* and *cifB* phylogenies are strongly congruent, which is compatible with a model where modification and rescue factors must be matched (Lindsey et al. 2018). In contrast, the *cif* gene and *Wolbachia* phylogenies are incongruent indicating that these genes are often transferred between *Wolbachia* genomes (Lindsey et al. 2018). CI genes are also commonly associated with prophage sequences on the *Wolbachia* chromosome, suggesting that phage-mediated transfer may underlie their mobility (Lepage et al. 2017; Lindsey et al. 2018). Additionally, *Wolbachia* genomes often contain multiple pairs of the *cifA* and *cifB* genes, which may explain complex patterns of bidirectional incompatibility (Bonneau et al. 2018). Finally, homologs of *cifA* and *cifB* frequently display signs of pseudogenization, carrying mutations that disrupt their open reading frame (ORF) or introduce premature stop codons (Asselin et al. 2018; Turelli et al. 2018; Meany et al. 2019). Therefore, the evolution of CI genes appears to be highly dynamic, being punctuated with acquisition events through horizontal transfer and losses by pseudogenization. However, the rate at which these events occur and to what extent they are governed by neutral processes or selection acting on the CI phenotype remains to be explored.

The identification of the *cifA* and *cifB* genes allows us to revisit theoretical predictions about the evolution of CI. A curious feature of CI is that it involves sperm being modified in males, and yet *Wolbachia* is not transmitted from males to future generations. This leads to the prediction that in randomly mating populations the ability to modify sperm is selectively neutral, so the genes involved can be lost by mutation and genetic drift (Turelli 1994; Hurst and McVean 1996). Once sperm modification has been lost, the rescue function will be free to suffer a similar fate (Turelli 1994; Hurst and McVean 1996). It has been suggested that the long-term maintenance of CI can be explained by kin selection in structured populations (*Wolbachia* in females benefits from related males inducing CI [Frank 1997]), but theoretical analyses suggest that this may be a weak force that acts only under specific circumstances (Haygood and Turelli 2009). This has led to the suggestion that CI may be maintained by a process of clade selection where CI is necessary for the horizontal transfer of *Wolbachia* to new species (Hurst and McVean 1996). There is an analogy between these models, as here *Wolbachia* populations are structured depending on which species they infect (clade selection model) as opposed to structure in space (kin selection model). Theory also predicts that multiple *Wolbachia* strains with different rescue and modification factors can invade populations (Frank 1998; Vautrin et al. 2007). A similar logic predicts that *Wolbachia* variants that acquire novel CI crossing types will invade infected populations provided they retain compatibility with the resident strain (Charlat et al. 2001). At the molecular level, this could be achieved by *Wolbachia* genomes accumulating *cifA–cifB* paralogs.

Here, we explore the evolution of *cifA* and *cifB* using publicly available and newly sequenced *Wolbachia* genomes. We found the genes in most *Wolbachia* genomes, often in multiple copies. Although the protein domains required for CI are widely conserved, the domain structure of divergent homologs found in some *Wolbachia* strains and related bacterial genera suggests that they may play a diverse range of molecular functions. The *cif* genes are lost on relatively short time scales, with the modification function usually being lost before the rescue function. This is compensated by pervasive horizontal transfer of functional *cif* genes between symbiont genomes, in many instances across long phylogenetic distances. Finally, recombination between *cif* genes is largely restricted to closely related homologs, supporting the hypothesis that genetic divergence leads to the diversification of CI compatibility types.

## Results

### New Genome Sequences of *Wolbachia* from *Drosophila*

**Fig. 1.**
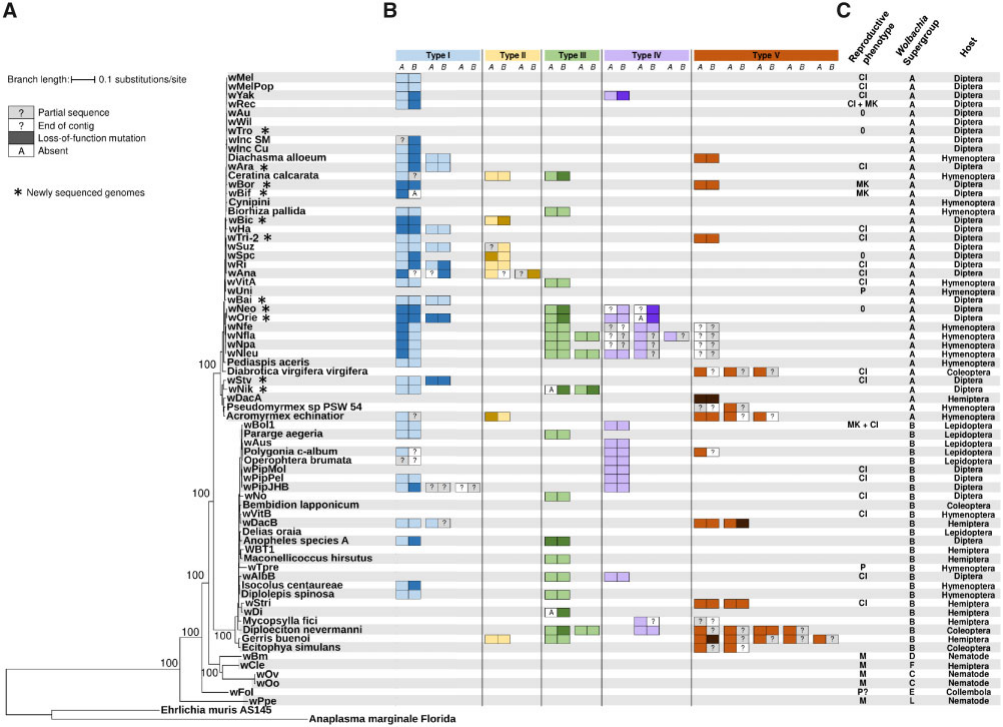
Distribution of *cifA* and *cifB* homologs across *Wolbachia* strains. (*A*) Maximum likelihood phylogeny of *Wolbachia* reconstructed from the concatenated sequences of 28 genes. Bootstrap values were estimated from 1,000 replicates. (*B*) *cifA* and *cifB* homologs. Each box represents a single gene and contiguous boxes indicate adjacent genes in the genome. Genes are organized according to Type, as defined from their genealogy (fig. 2 and supplementary fig. S1, Supplementary Material online). (*C*) Reproductive phenotypes (CI, cytoplasmic incompatibility; MK, male-killing; P, parthenogenesis; M, mutualism; 0, no reproductive phenotype), *Wolbachia* supergroups, and hosts in which the *Wolbachia* was found. The reproductive phenotype is not shown for strains for which conflicting phenotypic data exist (*w*Yak and *w*Suz).

We sequenced 11 *Wolbachia* genomes from 11 different *Drosophila* hosts, with mean sequencing depths ranging from 27× to 40× (table 1). Assembly sizes were within the range of typical *Wolbachia* genomes, with the genomes of the strains *w*Stv and *w*Nik forming a single scaffold. The number of near-universal, single-copy genes from the BUSCO proteobacteria database in the assemblies was similar to published reference genomes of *Wolbachia*, indicating that genomes are near complete (table 1; repeating the analysis on published genomes of *w*Mel, *w*Au, *w*Ha, *w*No, and *w*Ri yielded 181, 184, 183, 182, and 182 complete BUSCO genes, respectively). One of these strains, *w*Tri, has been previously sequenced by Turelli et al. (2018). Our sequence differed by 114 single nucleotide polymorphisms was more intact and contained an additional pair of *cif* genes. We named our strain *w*Tri-2. The newly sequenced strains all cluster within *Wolbachia* supergroup A, like most *Wolbachia* isolated from *Drosophila* hosts in our data set (fig. 1).

**Table 1.** Summary Statistics of Newly Sequenced *Wolbachia* Genomes.

Host	Strain	Mean Sequencing Depth	Genome Size (bp)^a^	GC Content (%)	*N* Scaffolds	*N* Contig	Scaffold N50	Contig N50	BUSCO Complete	BUSCO Fragment	BUSCO Missing
*Drosophila arawakana*	*w*Ara	30×	1,290,535	35.3	10	17	185,314	171,449	179	2	39
*Drosophila baimaii*	*w*Bai	40×	1,119,646	35.3	26	123	76,530	13,921	180	2	38
*Drosophila bicornuta*	*w*Bic	30×	1,177,727	35.1	24	34	71,402	49,062	182	1	37
*Drosophila bifasciata*	*w*Bif	33×	1,187,580	35.1	17	26	122,861	113,299	183	3	34
*Drosophila borealis*	*w*Bor	36×	1,210,092	35.3	16	39	146,013	52,110	183	2	35
*Drosophila neotestacea*	*w*Neo	40×	1,353,942	35.2	19	26	124,304	93,317	182	3	35
*Drosophila nikananu*	*w*Nik	27×	1,137,710	35.3	1	7	—	583,015	183	2	35
*Drosophila orientacea*	*w*Orie	27×	1,359,726	35.2	19	30	103,124	73,532	181	4	34
*Drosophila sturtevanti*	*w*Stv	30×	1,183,448	35.3	1	3	—	207,642	185	2	33
*Drosophila triauraria* ^b^	*w*Tri-2	33×	1,284,908	35.2	9	19	265,635	124,454	183	1	36
*Drosophila tropicalis*	*w*Tro	30×	1,214,296	35.2	13	17	106,850	67,971	184	2	34

a Ungapped genome size.

b This stock was identified in Mateos et al. (2006) and Miyake and Watada (2007) as *Drosophila quadraria* but Watada et al. (2011) later concluded that *quadraria* is a junior synonym for *triauraria*.

### The *Cif* Genes Are Widespread in the Genomes of *Wolbachia* and Related *Rickettsiales*

To examine the evolution of *cif* genes, we combined our newly sequenced genomes with published sequences (supplementary table S1, Supplementary Material online). This gave 71 *Wolbachia* genome sequences, in which we identified 129 and 128 homologs of *cifA* and *cifB*, respectively (fig. 1; supplementary table S2, Supplementary Material online). Synteny was highly conserved—in 115 cases (∼89%) *cifA* was located immediately upstream of *cifB* (fig. 1). A single *cifA* homolog and three *cifB* homologs broke this pattern and were present without their partner. Interestingly, all four of these genes carry mutations that disrupt their ORF suggesting that they are pseudogenes. The remaining genes not found in pairs were located at the end of contigs and/or were partially sequenced, preventing us from drawing conclusions about synteny. As the operon status of these genes is disputed (Beckmann, Bonneau, et al. 2019; Shropshire et al. 2019), we will refer to the 115 syntenic pairs of genes as *cifA*–c*ifB* genes.

Seventy-three percent (52/71) of *Wolbachia* strains carry at least one homolog of *cifA* or *cifB* (fig. 1), and almost half the *Wolbachia* genomes carry multiple *cifA*–c*ifB* homologs (35/71). The largest number was in the strains infecting *Diploeciton nevermanni* and *Gerris buenoi*, both of which had seven syntenic *cifA*–c*ifB* genes (fig. 1). The counts of gene pairs across strains did not differ from a Poisson distribution (Cameron–Trivedi test for Poisson equidispersion on per strain counts of intact syntenic *cifA*–c*ifB* genes in supergroups A and B: *z *=* *1.38, *P *=* *0.16 [Cameron and Trivedi 1990]).

**Fig. 2.**
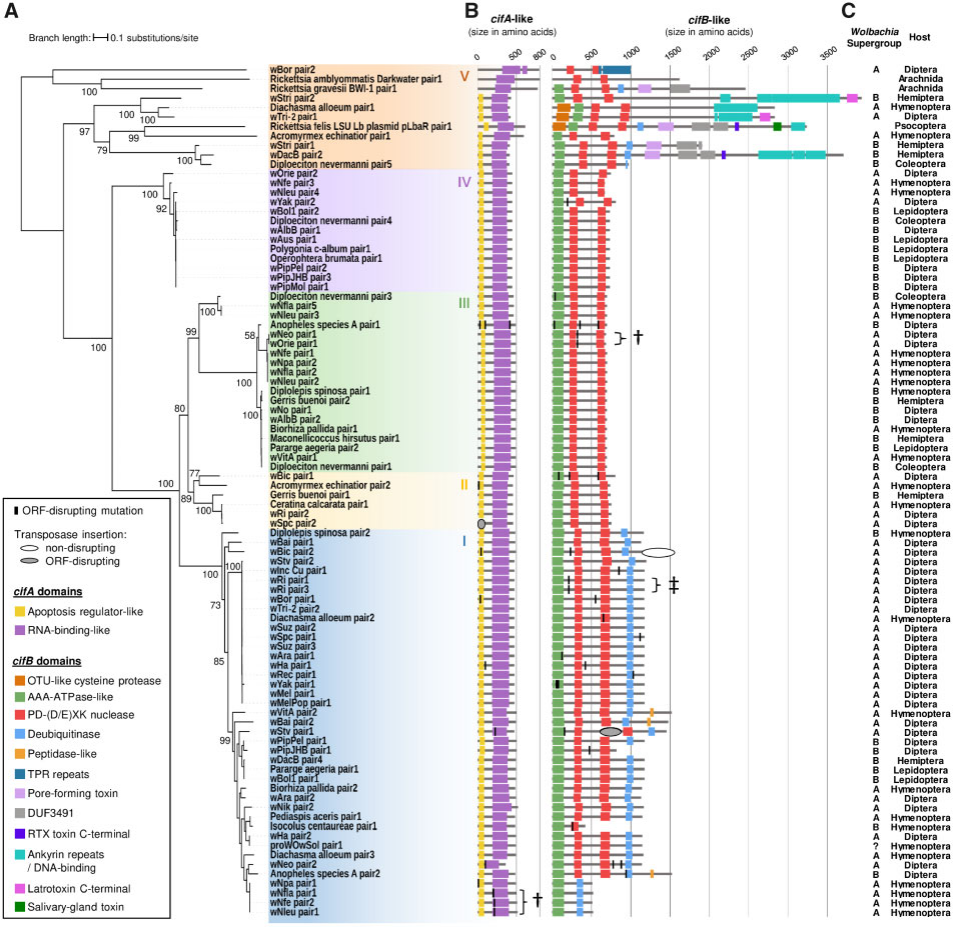
Phylogeny and protein domain architecture of *cifA* and *cifB*. (*A*) Maximum likelihood tree of concatenated *cifA* and *cifB* nucleotide sequences. Partially sequenced *cif* homologs were excluded. The tree is midpoint rooted. Bootstrap values were estimated from 1,000 replicates. (*B*) Protein domains. Mutations that disrupt the ORF are indicated by a vertical bar. Symbols indicate where the same ORF-disrupting mutation is found in two homologs due to speciation (†) or duplication (‡) events. All domains had a HHpred probability of being true positives >75% in at least one sequence. The suffix “-like” at the end of the domain name indicates that there were no sequences where the probability of the domain being true was >95%. Details of the domains are in supplementary table S5, Supplementary Material online. (*C*) *Wolbachia* supergroups and arthropod hosts from which the *cif* sequences were isolated.

To examine the distribution of *cifA*–c*ifB* genes across bacterial strains, we reconstructed the *Wolbachia* phylogeny using a set of 28 single-copy genes that were present in over 95% of the genomes. The *cifA* and *cifB* genes are widespread across the *Wolbachia* supergroups A and B, which contain most of the genomes analyzed (fig. 1). Six symbiont strains belong to other *Wolbachia* supergroups, and none of their genomes contained *cifA* or *cifB.* However, syntenic *cifA*–c*ifB* genes were identified in the genomes of *Rickettsia gravesii, R. amblyommatis*, and *Occidentia massiliensis* which infect ticks, and in a plasmid found in *R. felis* strain *LSU-Lb*, which infects the booklouse *Liposcelis bostrychophila* (fig. 2 and supplementary fig. S1 and table S2, Supplementary Material online).

### The *Cif* Genes Are Associated with CI

To examine the association between *cif* genes and phenotype, we compiled a list of the effects that each *Wolbachia* strain has on host reproduction (fig. 1*C*, supplementary table S1, Supplementary Material online). We found that there was a significant association between the presence of *cifA–cifB* genes (without ORF-disrupting mutations) and published reports of a strain inducing CI (table 2; Fisher’s exact test: *P *<* *0.0001). The only case of CI-inducing strain without these genes was *w*VitB; however, this may be an omission from the genome sequence. Indeed, we found what appeared to be fragments of *cifA* and *cifB* on very small contigs in the *w*VitB genome assembly. Another CI-inducing strain, *w*Rec, carries a syntenic *cifA*–c*ifB* pair where *cifB* has a frameshift mutation toward its 3′-end, downstream of predicted protein domains known to have a role in sperm modification (fig. 2, supplementary table S2, Supplementary Material online). Although we counted this as a pseudogene, it is possible that it encodes a functional protein. Finally, *w*Yak induces weak CI (Cooper et al. 2017) and both copies of *cifB* in the genome contain stop codons (Cooper et al. 2019; fig. 2).

**Table 2.** Association between CI and the Presence of *cifA–cifB* Genes in *Wolbachia* Genomes.

	CI^a^	
*cifA–cifB* ^b^	Yes	No
Present	14	1
Absent	1	10
Pseudogenes only	2	3

a *Wolbachia* strains for which there is no phenotypic information available or there are contradictory reports in the literature (*w*Suz) were discarded.

b “Present” stands for *cif* genes without loss-of-function mutations; partially sequenced *cifA–cifB* pairs were discarded. *w*Ana and the strain found in *Diabrotica virgifera virgifera* induce CI but are excluded from the table as they only have partially sequenced *cif* genes, preventing us from inferring their pseudogenization status.

There were 14 strains that are reported not to induce CI (table 2). Among them, the strain *w*Bor was the only one to carry a pair of *cifA*–c*ifB* genes without ORF-disrupting mutations, although this strain is only known to induce male-killing. As described below, both of these genes lack domains that are conserved across all CI-inducing strains. Among the other non-CI strains, the genes were absent from two *Wolbachia* strains that induce parthenogenesis (*w*Tpre and *w*Uni), one strain suspected to induce parthenogenesis (*w*Fol), two strains that do not induce CI or other clear phenotypic effects (*w*Au and *w*Tro), and five strains thought to be mutualists (*w*Cle in bed bugs and the four strains from nematodes) (fig. 1). Finally, the male-killing strain *w*Bif and two strains with no clear reproductive phenotype (*w*Spc and *w*Neo) were only found to carry *cif* gene pairs with ORF-disrupting mutations. Overall, the strong association between syntenic *cif* genes and the CI phenotype suggests a single evolutionary origin of this reproductive manipulation in *Wolbachia* (table 2).

### A New and Diverse Group of *Cif* Genes is Found in *Wolbachia* and Other *Rickettsiales*

Reconstruction of the *cifA*–*cifB* gene tree revealed new diversity among these genes. Although most sequences fell into four clades that have been named Types I–IV, the genes from two *Rickettsia* species, a *Rickettsia* plasmid, *O. massiliensis* and several *Wolbachia* strains were basal to these four types on the midpoint rooted tree (fig. 2; supplementary fig. S1, Supplementary Material online). The divergence among these sequences is frequently greater than between Types I–IV, and we are unable to root the phylogeny with high confidence without a reliable outgroup. However, midpoint rooting suggests that this may be a paraphyletic group with the other *cif* genes nested within it. We propose to call this diverse assemblage of *cifA–cifB* homologs “Type V.”

We found multiple protein domains, including the *cifB* nuclease and deubiquitinase domains known to be involved in sperm modification (see below for a detailed description of protein domain conservation). Type V *cifB* genes tend to be longer and possess a diverse array of domains, suggesting that they may perform a variety of molecular functions (fig. 2; supplementary fig. S1*B*, Supplementary Material online). Among these genes we identified toxin domains (Latrotoxin, RTX, pore-forming, and salivary-gland toxins), a protease domain (OTU-like cysteine protease), and domains involved in protein–protein interactions (tetratricopeptide and ankyrin repeats). The ankyrin repeat domain also shows strong similarities with DNA-binding proteins (probabilities ∼100% in the HHpred search).

All five *cifA–cifB* types are associated with CI. Type I genes from *w*Mel and *w*Pip, and Type IV genes from *w*Pip have been experimentally linked to CI (Beckmann et al. 2017; Lepage et al. 2017; Chen et al. 2019). Additionally, we found CI-inducing strains such as *w*No and *w*Stri that carry only Type III or Type V genes. Finally, in the CI-inducing strain *w*Ri the only *cifA–cifB* genes without signs of pseudogenization belong to Type II.

### The *cifA* and *cifB* Genes Codiverge with Recombination Restricted to Closely Related Genes

**Fig. 3.**
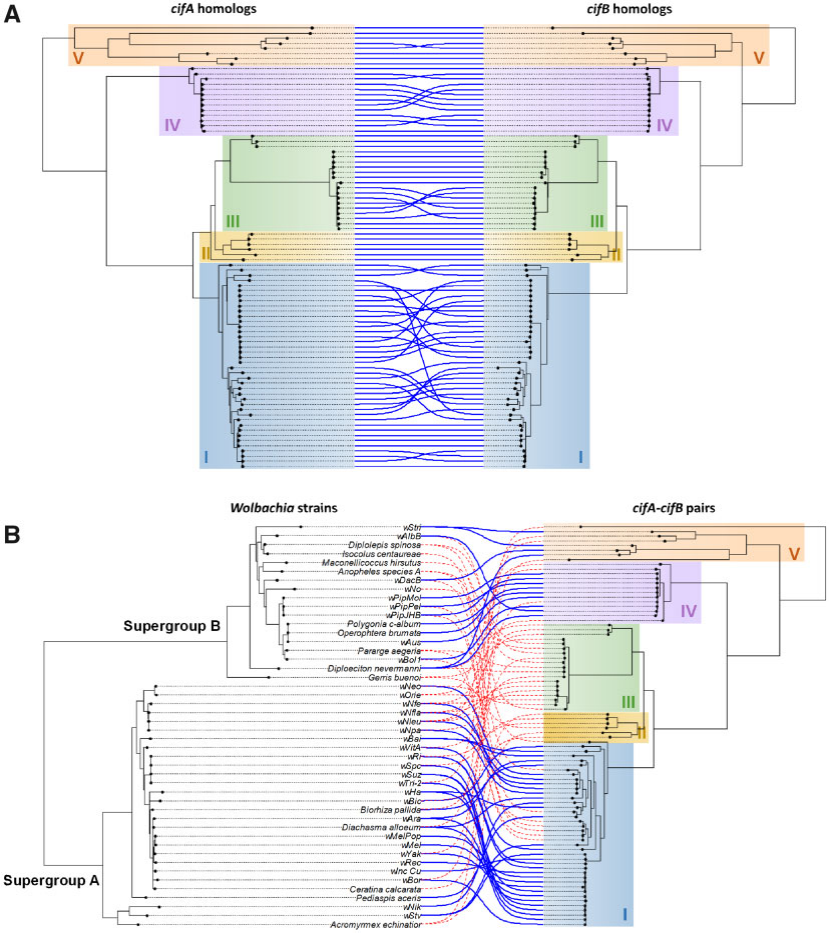
Phylogenetic congruence between *cif* genes and *Wolbachia* strains. (*A*) Cophylogeny of syntenic *cifA* and *cifB* genes. (*B*) Cophylogeny of *Wolbachia* genomes and the *cifA–cifB* genes. Links between phylogenies indicate (*A*) syntenic genes and (*B*) *Wolbachia* strain–*cif* gene associations, respectively. Blue links have a significant contribution to the global cophylogenetic signal in the Parafit test and red links do not.

The *cifA* and *cifB* proteins bind each other, and in a comparison of two *Wolbachia* strains the proteins encoded by syntenic pairs of genes bound more strongly than heterologous proteins (Beckmann et al. 2017; Chen et al. 2019). This led to the suggestion that coevolution of binding affinities between the proteins could underlie the divergence of CI crossing types (Beckmann, Bonneau, et al. 2019). Consistent with this and in agreement with earlier studies (Lepage et al. 2017; Lindsey et al. 2018), syntenic *cifA* and *cifB* genes show strong phylogenetic congruence (Mantel test *P*-value < 0.0001; supplementary fig. S2*A* and *B*, Supplementary Material online; fig. 3*A*). Strikingly, there is no case where recombination has brought together *cifA* and *cifB* genes from different Types (fig. 3*A*). Nonetheless, the two trees are not identical. Using multiple approaches to recombination detection on the concatenated alignment of *cifA* and *cifB*, we identified 83 well-supported recombination events (supplementary table S4, Supplementary Material online; note that some events may have been counted multiple times). Manual inspection of the sequences frequently revealed clear recombination breakpoints (supplementary fig. S3*A*–*C*, Supplementary Material online). However, all but one of these events involved sequences of the same Type (82/83 events; supplementary table S4, Supplementary Material online). This pattern of recombination tending to occur between closely related sequences was strongly supported, as the mean genetic distance between inferred parental sequences was significantly lower than expected by chance (supplementary fig. S4, Supplementary Material online). Manual inspection of the only recombination event involving parental sequences belonging to different types revealed no clear breakpoint in the recombinant sequence, suggesting that this event could be a false-positive created from low-quality alignment between highly divergent homologs (supplementary fig. S3*D*, Supplementary Material online). Together, these results indicate that *cifA* and *cifB* recombination is largely restricted to closely related sequences, perhaps because their binding affinities have coevolved.

### A Conserved Protein Domain Architecture Is Associated with CI

To gain insights into the molecular basis of CI and its evolution, we used a comparative approach to identify the protein domains associated with the trait. A complication is that closely related domains may be annotated in some sequences but not others depending on whether they meet an arbitrary significance threshold. To avoid this, we first searched for domains using conventional tools, and then used these sequences to create a *Wolbachia*-specific Hidden Markov Model (HMM) profile for each domain that was used to repeat the search.

The induction of CI by the Type IV *cifB* paralog in wPip (*cinB*) requires DNAse activity due to its PD-(D/E)XK nuclease domains (Chen et al. 2019). However, the Type I *cifB* paralog in *w*Pip (*cidB*) has lost the catalytic residues associated with DNase activity and instead CI requires a deubiquitinase domain that functions to deconjugate ubiquitin from proteins (Beckmann et al. 2017; Beckmann, Sharma, et al. 2019). We found that the two PD-(D/E)XK nuclease domains were highly conserved across the *cifB* tree (fig. 2; supplementary fig. S1*B* and table S5, Supplementary Material online). As is normally the case, these domains were associated with a 5′ AAA-ATPase domain (Knizewski et al. 2007). The catalytic D–E–K residues were conserved throughout *cifB* evolution until they were lost in the common ancestor of the Type I genes (supplementary fig. S6, Supplementary Material online, Lindsey et al. 2018; Chen et al. 2019). This coincided with the Type I genes acquiring the deubiquitinase domain, and this domain is conserved across Type I. This suggests that the molecular basis of CI has been conserved across the Type II, III, and IV genes, and then changed (Chen et al. 2019) in the ancestor of the Type I sequences. Interestingly, the deubiquitinase domain is also present in some but not all Type V *cifB* homologs, suggesting that these might induce CI through a mechanism similar to that of the Type I genes (fig. 2; supplementary fig. S1*B* and table S5, Supplementary Material online). The sparse distribution of the deubiquitinase domain raises questions about its origin. A phylogenetic reconstruction of the deubiquitinase domain’s amino acid sequence grouped Type I and V deubiquitinase domains as two monophyletic clades (supplementary fig. S7, Supplementary Material online), so the domain does not appear to have been exchanged by recombination. Instead, the deubiquitinase domain must either have been acquired independently by the Type I and V genes or have been present in the common ancestor of the *cif* genes and subsequently lost multiple times.

The *cifA* gene has a more conserved domain structure than *cifB* (fig. 2; supplementary fig. S1*A* and table S5, Supplementary Material online). The two domains that we identified were annotated as functioning in apoptosis regulation and RNA-binding, but we caution that the support for these annotations being true positives was <85%. There are six *cifA* genes that have lost the apoptosis regulator-like domain (including two putative pseudogenes), and none of these is known to induce CI (supplementary fig. S1*A*, Supplementary Material online). If some of these strains were found to induce CI, it would be of interest to test whether they can also rescue this effect, to assess whether this domain is required for the rescue activity of *cifA* proteins.

### The *Cif* Genes Frequently Transfer between Distantly Related *Wolbachia* Genomes and Insect Hosts

The *cif* genes are often found in the vicinity of prophage genes, a cluster of genes known to undergo intense genomic rearrangements and horizontal transfer between *Wolbachia* genomes (Bordenstein and Wernegreen 2004). The link between *cif* genes and mobile genetic elements is reinforced by the presence of the genes on a plasmid found in *Rickettsia.* There are numerous cases of the genes being exchanged between distantly related bacterial hosts, including multiple instances of transfer between the *Wolbachia* supergroups A and B (fig. 3*B*). This suggests that horizontal transfer of *cif* genes between distantly related symbionts is common, sometimes even crossing the bacterial genus boundaries as exemplified by the presence of *cifA–cifB* homologs in *Rickettsia* genomes and plasmids (fig. 2; supplementary fig. S1, Supplementary Material online). Nonetheless, there is partial congruence between the phylogenies of *Wolbachia* and their *cif* genes (Mantel test *P*-value < 0.0001; fig. 3*B*;supplementary fig. S2*C* and *D*, Supplementary Material online). This could result from *cif* genes being maintained for long enough to be coinherited with the *Wolbachia* genome during speciation events or the genes frequently transferring between closely related bacterial strains.

As the *cifA* and *cifB* proteins interact with targets in the arthropod host, their distribution may be constrained by the arthropod phylogeny rather than the *Wolbachia* phylogeny. Despite the *cifA* and *cifB* genes frequently transferring between distantly related arthropods, there is a highly significant tendency for closely related *cif* genes to be found in the same host order (Mantel test comparing *cifA–cifB* pairwise distances and insect orders: *P*-value < 0.0001; supplementary fig. S2*E* and *F*, Supplementary Material online). However, we would caution that this association could be the result of the *Wolbachia* phylogeny being correlated with the arthropod phylogeny (Mantel test comparing *Wolbachia* pairwise distances and insect orders: *P*-value < 0.0001; supplementary fig. S2*G* and *H*, Supplementary Material online) and these effects are difficult to disentangle.

### Pseudogenes Are Common and the Loss of Sperm Modification Usually Predates the Loss of the Rescue

In randomly mating populations there is no selection for CI-inducing *Wolbachia* to modify sperm (Turelli 1994), so a gene whose only function is sperm modification (*cifB*) is predicted to eventually lose its function. Once this occurs there is no selection to maintain the rescue function, so *cifA* can also be lost. Excluding partially sequenced genes, putative loss-of-function mutations were found in 12.1% of *cifA* sequences (15/124) and 27.8% of *cifB* sequences (27/97), suggesting that the modification of sperm function is lost more frequently than the rescue function (supplementary fig. S1*A* and *B*, Supplementary Material online; Fisher’s exact test: *P *=* *0.02). The fixation rate of loss-of-function mutations per site was not significantly different between the two genes (*cifA*: 2.4 × 10^−4^ mutations/amino acid; *cifB*: 3 × 10^−4^ mutations/amino acid; Fisher’s exact test: *P *=* *0.64).

To examine the order in which *cifA* and *cifB* become pseudogenes, we visually inspected the 87 fully sequenced *cifA–cifB* pairs. We identified 38 independent mutational events that cause the ORF to be disrupted (i.e., not double-counting mutations inherited through speciation or duplication events; fig. 2). These putative loss-of-function mutations include single-base pair substitutions introducing premature stop codons, indels producing frameshifts, insertion of transposable elements (*w*Stv pair 1), and short inversions (*w*Yak pair 1) (supplementary table S2, Supplementary Material online). The majority of *cifA–cifB* gene pairs shows no signs of pseudogenization (63/87), whereas six carry ORF-disrupting mutations in both genes. In cases where just one gene carried a mutation, it was more often *cifB* than *cifA* (14 vs. 4 instances; fig. 2; binomial test: *P *=* *0.03). This suggests that sperm modification function is usually being lost before the rescue function. Interestingly, in two cases where only *cifA* appears to be pseudogenized (*Wolbachia* in *Nomada* bees), the *cifB* genes are ∼50% shorter than their close relatives and lack the nuclease domains conserved in all other *cifB* genes (fig. 2). Therefore, these *cifB* genes may be nonfunctional despite the absence of ORF-disrupting mutations. In a third case, *cifA* is pseudogenized in *w*Spc, a strain that does not modify sperm in its host *D. subpulchrella* despite carrying an intact *cifB* gene, meaning that there should be no selection acting to maintain the rescue function in this strain (fig. 1).

Once the CI genes lose their function, it has been predicted that the *Wolbachia* infection will also be lost from the population. This should happen unless other phenotypes such as the provision of fitness benefits to the host are maintaining the symbiont, or the *Wolbachia* genomes harbor additional *cif* genes that are still functional. If the infection is maintained, nonfunctional CI genes may accumulate further loss-of-function mutations or be eliminated from the *Wolbachia* genomes, for instance, by excision of the prophage harboring the CI genes. By looking at the phylogenetic distribution of putative loss-of-function mutations, it is possible to infer whether nonfunctional *cif* genes slowly degenerate or are quickly eliminated. The majority of loss-of function mutations is located on the terminal branches of the CI gene phylogenies (fig. 2; supplementary fig. S1*A* and *B*, Supplementary Material online). The only exceptions are a few mutations coinherited between closely related *Wolbachia* strains (*w*Neo/*w*Orie; *Wolbachia* from *Nomada* bees) or through a putative duplication in the *w*Ri genome (pairs 1 and 3 are identical). This suggests that pseudogenized *cif* genes are rarely horizontally transmitted between *Wolbachia* genomes or maintained for long enough to be inherited through speciation events.

### Closely Related *Cif* Genes Commonly Coexist in the Same Genome

CI favors female hosts that have the greatest reproductive compatibility with males in the population. Therefore, *Wolbachia* strains that induce a new CI crossing type can invade *Wolbachia-*infected populations provided they are compatible with the resident strain (Charlat et al. 2001). Such a mutant could arise if *Wolbachia* acquires an additional pair of *cif* genes, and this may explain why *Wolbachia* genomes commonly harbor multiple *cif* paralogs. However, additional *cif* genes should only spread if they induce a different crossing type from the genes already present in the genome. If closely related genes confer the same crossing type, then this would mean that closely related *cif* homologs are unlikely to be found within the same genome. However, we found no support for this as in our data set—putatively functional *cif* homologs in the same genome have very similar levels of divergence to *cif* homologs found in different genomes (supplementary fig. S5, Supplementary Material online). A similar argument applies to the loss of *cif* genes—if a genome contains paralogs that induce the same crossing type then one of the paralogs could be lost by mutation. However, again there is no evidence for this process. Looking within genomes, the genetic distance between intact *cif* genes and putative pseudogenes was similar to the genetic distance between pairs of functional genes (genetic distance calculated from fig. 2: 1.15 and 1.28, respectively).

## Discussion

CI is the most commonly observed reproductive manipulation induced by *Wolbachia*, and its evolution has been investigated for over 60 years through phenotypic experiments (Laven 1957; Sinkins et al. 1995; Charlat et al. 2003; Zabalou et al. 2008) and evolutionary models (Caspari and Watson 1959; Turelli 1994; Hurst and McVean 1996; Vautrin et al. 2007). The discovery of the genes underlying CI now makes it possible to reconstruct the trait’s evolution at the molecular level and infer the selection pressures acting on CI using the tools of molecular evolution. Here we analyzed 71 *Wolbachia* genome sequences to investigate the importance of recombination, pseudogenization, and horizontal gene transfer in the evolution of CI.

The *cif* genes are widespread across the *Rickettsiales*. Our large data set supported the observation of Lindsey et al. (2018) that *cif* genes are common in *Wolbachia* supergroups A and B. These supergroups contain most of the *Wolbachia* strains that have been described, and here the genes are tightly linked to the CI phenotype. The genes were absent from a small sample of *Wolbachia* strains from other supergroups. This may explain why, to our knowledge, there are no reports of strains from these supergroups inducing CI. However, divergent Type V *cif* genes are found in other *Rickettsiales*, and here their phenotypic effects are uncertain (Gillespie et al. 2018). Gillespie et al. (2018) proposed that *cif* homologs found in *Rickettsia* may induce other reproductive phenotypes. For instance, *R. felis* strain LSU-Lb, which carries Type V *cif* genes on its plasmid, infects a parthenogenetic insect. Accurate rooting of the *cif* gene tree together with functional characterization of these genes will be needed to confirm whether the ancestral function of these genes was to induce CI. However, the presence of a CI-inducing *Wolbachia* strain that has only Type V genes suggests that CI evolved very early in the evolution of *cif* genes.

It has long been observed that crosses between insects carrying different *Wolbachia* strains are frequently incompatible, suggesting that modification and rescue factors must be matched to produce viable offspring. A possible molecular cause of this phenomenon comes from the observation that *cifA* and *cifB* bind each other (Beckmann et al. 2017; Chen et al. 2019), and so binding affinities may be greatest between coevolved genes. If this is the case, then there would be strong selection against recombination between the genes as this could generate symbionts that are unable to rescue crosses with infected females (Charlat et al. 2001). Furthermore, even if the symbiont retained the ability to rescue the cross, for example, if genes are swapped among paralogous pairs within the genome, the pair of genes might be eliminated in the long term if it generated self-incompatibility when transferred to a new genome. Despite this, clear evidence of recombination has been observed among *cif* genes from *w*Pip, which infects *C. pipiens* mosquitoes (Bonneau et al. 2018). *w*Pip strains can carry multiple copies of Type I *cif* genes, and recombination between closely related paralogs correlates with different CI crossing types. Whether recombination itself created these incompatibilities or whether crossing types arose due to sequence divergence following recombination is unknown. We found that recombination is frequent across the *cif* gene phylogeny, but it almost exclusively occurs between related syntenic *cifA–cifB* gene pairs within the same Type. As *Wolbachia* genomes often carry divergent *cif* homologs, the absence of recombination between these genes is compatible with recombination being constrained by selection. This can be explained by the coevolution of binding affinities between the proteins encoded by *cifA* and their cognate *cifB* gene.

The evolution of sperm modification by *Wolbachia* poses an evolutionary puzzle—the trait is only expressed in males and yet symbionts in males are not passed on to the next generation. In randomly mating populations, it will be at best selectively neutral (Prout 1994; Turelli 1994). Although kin selection can act to maintain CI in structured populations (Frank 1997), this is a weak force that operates only under specific circumstances (Haygood and Turelli 2009). Therefore, theory predicts that a gene involved exclusively in sperm modification, such as *cifB*, will accumulate loss-of-function mutations. Once these have been fixed within a population, the gene required for the rescue function (*cifA*) will then degenerate by mutation. It has already been reported that *cif* genes often carry putative loss-of-function mutations (Asselin et al. 2018; Lindsey et al. 2018; Cooper et al. 2019; Meany et al. 2019). We found *cifB* carries loss-of-function mutations more often than *cifA*, and that *cifA* generally acquires such mutations after *cifB*. This confirms a key prediction of theory and supports the hypothesis that lack of selection to maintain sperm modification may lead to the loss of CI in some populations (Turelli 1994; Hurst and McVean 1996). Although evolutionary predictions were based on a mechanistic model that assumes sperm modification and rescue were encoded by different genes, the same pattern of gene loss would be expected if both *cifA* and *cifB* are required to modify sperm (Shropshire and Bordenstein 2019). This is because a mutant that lost *cifA*, and therefore the ability to both induce and rescue CI, would initially be rare in a population of CI-inducing symbionts and should be counterselected. By contrast, mutating *cifB* will only cause the loss of sperm modification and will therefore not be exposed to selection. Therefore, even in the two-by-one mechanistic model, the spread of a symbiont carrying a pseudogenized *cifA* and a functional *cifB* is unlikely. Nonetheless, how the two-by-one model affects details of the dynamics, such as the effects of population structure, remains to be explored theoretically.

The degeneration of *cif* genes could also occur if the CI phenotype is lost first, for instance, due to the host evolving to suppress the trait (Koehncke et al. 2009), leaving the genes free to degenerate by mutation. This is plausible as artificial transfers of *Wolbachia* between species have shown that host genetic background can affect the expression of CI (Poinsot et al. 1998; Jaenike 2007; Zabalou et al. 2008). As this model makes no predictions about the order of gene loss, it may explain why we found at least one *Wolbachia* strain that does not induce any reproductive incompatibility and whose genome contains a *cifA* pseudogene alongside an intact *cifB.* As *cifB* is typically longer than *cifA*, even under this model of evolution *cifB* may tend to acquire loss-of-function mutations first. Indeed, the number of loss-of-function mutations per amino acid is similar between *cifA* and *cifB*, which is compatible with the two genes evolving under similar selective pressures. However, this is a rather weak test as after a loss-of-function mutation in *cifB*, under any evolutionary model both genes are free to degenerate at the same rate. Therefore, to separate these models it is necessary to know whether strains carrying *cifB* pseudogenes alongside functional *cifA* genes are found in hosts that can express CI. An example is *w*Ri. This strain induces CI, and its genome contains an intact pair of *cif* genes alongside two distantly related pairs of genes where *cifB* is a pseudogene. Another apparent example of this is *w*Yak, which has two copies of *cifB* in its genome, both of which contain internal stop codons (fig. 1; Cooper et al. 2019). When its natural host *D. yakuba* was experimentally infected with a different *Wolbachia* strain, it induced strong CI (Zabalou et al. 2004). However, the recent discovery that *w*Yak itself induces weak CI means that it is unclear whether the mutations in the two *cifB* genes reduced the ability of *w*Yak to modify sperm (Cooper et al. 2017). Together these examples provide tentative support for the model that sperm modification may degenerate by mutation, even in hosts expressing CI.

In the absence of other phenotypic effects on the host, theory predicts that the loss-of-sperm modification will lead to the loss of *Wolbachia* from the host population (Turelli 1994; Hurst and McVean 1996). We found that the majority of loss-of-function mutations appears to have occurred recently. Firstly, these mutations tend to be on terminal branches of the gene tree. Secondly, pseudogenes rarely accumulate many loss-of-function mutations. Although this suggests that *cifA* and *cifB* pseudogenes rarely codiverge with the *Wolbachia* genome for long periods, it is unclear whether this results from the loss of the *Wolbachia* infection or through the genes being deleted from the genome, for instance, by phage excision. Since the publication of theoretical studies on CI evolution (Turelli 1994; Hurst and McVean 1996), it has become increasingly clear that many *Wolbachia* strains can persist in host populations without inducing any reproductive manipulation by providing fitness benefits (Dedeine et al. 2001; Taylor et al. 2005; Hosokawa et al. 2010; Kriesner et al. 2013; Martinez et al. 2014; Kriesner and Hoffmann 2018). Therefore, it remains to be demonstrated whether the degeneration of *cif* genes causes the loss of *Wolbachia* from populations.

The frequent loss of *cif* genes raises a paradox when trying to explain the high prevalence of CI across *Wolbachia* strains. Hurst and McVean (1996) argued that CI-inducing *Wolbachia* infections were more likely to transfer horizontally between host species, so symbiont strains that induce CI are more likely to persist over an evolutionary timescale. Consistent with this process of clade selection*, Wolbachia* frequently jumps between host species (Zhou et al. 1998; Vavre et al. 1999; Baldo et al. 2008). Our observations suggest that clade selection may also be acting at the level of the *cif* genes as *Wolbachia* genomes appear to be frequently recolonized by *cif* genes from other symbionts. The pervasive horizontal transfer of *cif* genes may allow them to evade inevitable extinction within symbiont lineages by escaping into a new symbiont population. This process is analogous to the evolution of transposable elements, which frequently go extinct within host species, but persist long term by jumping into new species (Schaack et al. 2010).

Horizontal transfer of *cif* genes occurs frequently and sometimes over large phylogenetic distances, sometimes even crossing the bacterial genus boundaries. High rates of horizontal transfer likely result from the *cif* genes being associated with mobile genetic elements, such as WO prophage sequences, transposons, and plasmids (Gillespie et al. 2018; Lindsey et al. 2018; Cooper et al. 2019). Interestingly, Lepage et al. (2017) found no significant association between the phylogenies of *cif* genes and genes found in the structural module of phages. Prophage regions often rearrange (Bordenstein and Wernegreen 2004) which likely explains this pattern. However, this does not mean that phages are not a major route of horizontal transmission of *cif* genes, and the association between *cif* genes and mobile genetic elements supports the idea that the ability to move horizontally between genomes may be an important adaptation of these elements. Indeed, *cif* genes that lose their association with mobile elements may ultimately go extinct as they would be less likely to undergo horizontal transfer. As argued by Beckmann, Bonneau, et al. (2019), in some cases CI may be best viewed as an adaptation of mobile genetic elements to spread within *Wolbachia* populations.

*Wolbachia* genomes often carry multiple *cifA–cifB* gene pairs. This is analogous to the frequent occurrence of multiple *Wolbachia* strains within the same host individuals. Both experimental and theoretical studies have demonstrated that such multiple infections can invade host populations provided the strains carry different modification and rescue factors (Sinkins et al. 1995; Rousset et al. 1999). This is because females harboring additional *Wolbachia* strains will be compatible with all males in the population. An equivalent process will promote the invasion and maintenance of *cif* paralogs within the same genome, provided that they encode bidirectionally incompatible modification and rescue factors. If bidirectionally incompatibility evolves gradually, this hypothesis predicts that paralogous *cif* genes within a genome might be distantly related. However, we found no evidence for this, and frequently paralogs within the same genome are closely related. We would argue that this does not mean that we should discount the hypothesis that *cif* paralogs accumulate within the same genome because they encode bidirectionally incompatible CI factors. In particular, our analysis assumes that genetic divergence can be used as a proxy for the divergence of crossing types and this may not be the case. For example, the evolution of new crossing types could occur following *cif* gene duplication events (Beckmann, Bonneau, et al. 2019), meaning that a single genome could harbor closely related paralogs that encode incompatible crossing types, as observed in the *w*Pip-*Culex* system described above (Bonneau et al. 2018). Although sequence divergence following duplication is thought to have led to new compatibility types (Beckmann, Bonneau, et al. 2019), an open question is whether carrying multiple identical copies of a *cif* gene pair might be sufficient to produce new crossing types, perhaps by “dose effects.”

There is both direct and indirect evidence that homologs from all the main *cif* gene types can induce CI, and analysis of protein domains suggests that the molecular basis of CI is conserved. The *cifA* domain structure varies little across the gene family. All *cifB* genes associated with CI had one of the domains that has been experimentally linked to CI—a functional PD-(D/E)XK nuclease domain or a deubiquitinase domain (Beckmann et al. 2017; Chen et al. 2019). As previously reported, Type I *cifB* genes lack the catalytic residues in their PD-(D/E)XK nuclease domains, and instead have the deubiquitinase domain (Beckmann et al. 2017). Unexpectedly, this domain is also present in some divergent Type V sequences, making its evolutionary origins unclear. Alongside these conserved core domains, we found a diverse range of other *cifB* domains, notably in the long Type V genes. It is of interest whether these additional *cifB* domains are associated with CI. Given the relative homogeneity of *cifA* domains and the similarities of these additional *cifB* domains with known toxins and eukaryotic-like ankyrin proteins, it is tempting to hypothesize that they are linked to the modification function by interacting with the host rather than the binding to *cifA*. It would be interesting to test whether these domains allow manipulation of reproduction across a broader host range. Interestingly, one of these domains, the ovarian tumor domain (OTU), is also found in a toxin involved in male-killing induced by the symbiont *Spiroplasma poulsonii*, raising the possibility that some domains could be involved in multiple forms of reproductive manipulations.

In conclusion, our study illustrates the dynamic evolution of CI genes and highlights the high rates of gene loss and horizontal gene transfer. Further functional analysis will open new avenues of research and allow us to reconstruct the full evolutionary history of CI. In particular, the identification of the insect factors targeted by the sperm modification may soon allow us to study the coevolution of *cif* genes with the insect reproductive system (Beckmann, Sharma, et al. 2019). Finally, a deeper analysis of divergent *cif* homologs found outside *Wolbachia* should allow us to address questions around the deep evolutionary origin of CI and may reveal novel functions of these genes.

## Materials and Methods

### Sequencing of New *Wolbachia* Genomes

New *Wolbachia* genomes were obtained from 11 *Drosophila* species (listed in table 1) using the protocol described in Ellegaard et al. (2013). Briefly, *Wolbachia* cells were purified from 20 to 30 fly embryos that were dechorionated in bleach and homogenized in phosphate-buffered saline. The homogenate was then centrifuged, passed through 5- and 2.7-µm pore size filters and multiple-displacement amplification was performed directly on the bacterial pellet using the Repli-g midi kit (Qiagen) as in Ellegaard et al. (2013). The amplified DNA was finally cleaned using the QIAamp DNA mini kit prior to sequencing. From each DNA sample, 3-kb paired-end and 50-bp paired-end DNA libraries were prepared. These were multiplexed and sequenced on one plate of 454 Roche FLX (University of Cambridge, Department of Biochemistry, United Kingdom) and one lane of Illumina HiSeq2000 instruments (The Genome Analysis Centre, Norwich, United Kingdom), respectively.

454 and Illumina reads were used to perform hybrid de novo assemblies in Newbler v2.6 (454 Life Sciences Corp., Roche, Branford, CT). Non-*Wolbachia* contigs were then removed from each assembly by aligning the contigs to the *w*Mel reference genome (GenBank accession number: NC_002978.6) using Mauve v2.3.1 (Darling et al. 2004) and visual comparisons in the Artemis Comparison Tool (Carver et al. 2005). A BlastN analysis of the discarded contigs revealed positive matches with *Drosophila* mitochondrial and nuclear genomes, as well as with *Saccharomyces cerevisiae* (yeast used to collect the fly embryos), suggesting low levels of contamination during the DNA extraction process. Scaffolding was refined using SSPACE v2 (Boetzer et al. 2011) and gaps were filled with Gapfiller v1.11 (Boetzer and Pirovano 2012). Additionally, for strains *w*Stv, *w*Ara, and *w*Bor, Illumina reads were assembled separately using Abyss v1.3.5 (Simpson et al. 2009) and the contigs generated as well as the Illumina reads were mapped onto the hybrid assemblies using Consed (Gordon et al. 1998) in order to manually edit the scaffolds. Final assemblies were annotated as in Ellegaard *et al.* (2013) using a custom pipeline. In brief, gene and pseudogene predictions were performed using Prodigal (Hyatt et al. 2010) and GenePrimp (Pati et al. 2010), respectively. Domain prediction was done using hmmsearch implemented in pfam_scan.pl with the PFAM database (Bateman et al. 2002). Finally, annotations were manually edited through visual inspection in Artemis (Carver et al. 2012). The completeness of the assemblies was assessed using BUSCO v3 by searching the genomes against the near-universal, single-copy genes of the proteobacteria database (Simão et al. 2015).

### BLAST Search of CI Genes and Annotation

Previously identified *cif* gene homologs have been categorized into four phylogenetic clusters denominated as Type I–IV, as well as an uncharacterized type of more divergent homologs found in the *Wolbachia* strain *w*Stri (Lindsey et al. 2018). The presence of *cif* gene homologs in publicly available and newly sequenced *Wolbachia* genomes (supplementary table S1, Supplementary Material online) was searched with TBlastN (https://blast.ncbi.nlm.nih.gov/Blast.cgi; last accessed August 20, 2020) using *cifA* and *cifB* amino acid sequences representative of each CI type as queries (supplementary table S2, Supplementary Material online). Some of the genomes assembled in Pascar and Chandler (2018) were excluded from our analysis as they showed signs of multiple infections based on the assembly size, the sequencing coverage, and/or the presence of duplicated BUSCO reference genes. Additionally, when more than one genome were sequenced from the same host species and we found no evidence in the literature that they correspond to different *Wolbachia* strains, only one of them was included in the analysis. Default parameters and an *e*-value threshold of 0.05 were used. TBlastN hits across the *Wolbachia* genomes were then visually inspected in Artemis. Hits that were at least 40% of the length of the smallest query sequence and/or hits displaying the typical *cifA–cifB* synteny were considered as positive matches. Where sequences did not span the entirety of an ORF, they were manually extended to include the closest start and stop codons. The presence of stop codons and frameshifts within the reannotated sequences were interpreted as indicative of a putative pseudogenization event. DNA sequences were aligned with Clustal Omega using default parameters (https://www.ebi.ac.uk/Tools/msa/clustalo/; last accessed August 20, 2020), and putative “loss-of-function” mutations were examined by comparing closely related sequences and were defined as unique mutational events or, when found in more than one homolog, as coinherited.

Using BlastP online tool (https://blast.ncbi.nlm.nih.gov/Blast.cgi; last accessed August 20, 2020) with *cifA^wMel^* and *cifB^wMel^* amino acid sequences and the more divergent sequences found in *w*Stri as queries, we also found additional homologs in prophage WOSol (AGK87106 and AGK87078) and in non-*Wolbachia* taxa (Rickettsial plasmid genes pLbAR_36/38: WP_039595309.1/WP_081996388.1; *R. gravesii*: NZ_AWXL00000000.1; *R. amblyommatis*: GCA_000964995.1; *O. massiliensis*: CANJ01000001). These *cif* homologs were searched again and manually annotated as above from the original nucleotide sequences present in GenBank (https://www.ncbi.nlm.nih.gov/genbank/; last accessed August 20, 2020).

### CI Genes Phylogeny and Recombination

Following the manual reannotation, *cifA* and *cifB* DNA sequences were aligned separately based on their amino acid translations using TranslatorX (Abascal et al. 2010). The alignment program MAFFT implemented in the TranslatorX pipeline was used along with GBlocks in order to filter out weakly conserved regions from the alignment. The same was done for concatenated *cifA* and *cifB* sequences. PhyML v3.0 (Guindon et al. 2010) was used with the GTR GAMMA substitution model of evolution and 1,000 bootstrap replicates. All phylogenies were first reconstructed with all sequences, including partial ones (located at the end of genome contigs or showing bases called as Ns within their ORF) to classify the genes into phylogenetic types. Phylogenies were then rerun without partial sequences for the remaining analyses.

Recombination was analyzed using the recombination detection program RDP4 (Martin et al. 2015). The alignment of the concatenated *cifA* and *cifB* linear sequences was used to detect putative recombination events with the default parameters of the six methods implemented in RDP4 (RDP, GENECONV, Bootscan, MaxChi, Chimaera, and SiScan). Recombination events with a phylogenetic support and a Bonferroni-corrected *P*-value <0.05 with at least four of the detection methods were interpreted as reliable evidence of recombination. Out of 83 significant events, 22 were randomly selected and visually inspected to ensure that they were genuine. This was done by realigning the amino acid sequences of the putative recombinant and the two inferred parents.

### Prediction of Functional Domains

Protein domains were predicted for fully sequenced *cifA* and *cifB* homologs using the HHpred webserver (https://toolkit.tuebingen.mpg.de/#/tools/hhpred/ (last accessed August 20, 2020); Söding et al. 2005) with defaults parameters as in Lindsey et al. (2018). Premature stop codons were first removed manually from putative pseudogenes and DNA sequences translated into amino acid sequences. Amino acid sequences were then queried individually against the following databases: SCOPe70 (v.2.07), Pfam (v.32.0), SMART (v6.0), and COG/KOG (v1.0). Only hits with probabilities >75% in at least one *cif* homolog were considered as putative functional domains. In many cases, predicted domains on one sequence were not detected by HHpred on closely related homologs, although their presence could be suspected by visual inspection of the sequence alignments. In order to refine our domain search, we used representative amino acid sequences of each domain to build HMM profiles using hmmbuild implemented in HHMMER v3.2.1 (Eddy 2011). Representative domain selection was conducted by choosing domains distributed across the CI gene phylogenies, encompassing the maximum genetic divergence between reference homologs. Basically, one domain copy per phylogenetic type as well as all copies from non-*Wolbachia* taxa were chosen as references where available. When domains were missing from some types, a maximum of five domain copies were selected across the different types or all copies if there were fewer than five copies in total.

All CI homologs were then scanned using hmmscan and the HMM profile created from each functional domain with an *e*-value inclusion threshold of 0.0001. The amino acid sequences of hmmscan hits were extracted and reused to build new HMM profiles that were used to scan the CI genes again. Three search iterations were performed in this way which allowed us to retrieve many domains that HHpred failed to detect. Finally, domain coordinates were extracted and, in the case of putative pseudogenes, they were manually edited to take into account the presence of loss-of-function mutations in the original DNA sequences.

### *Wolbachia* Strain Phylogeny

Twenty-eight single copy genes that were present in >95% of the bacterial strains were identified using Phylosift v1.0.1 (Darling et al. 2014) (supplementary table S3, Supplementary Material online). The genomes of *Anaplasma marginale* (NC_012026.1) and *Ehrlichia muris* (NC_023063.1) were included as outgroups. For each genome, DNA sequences were concatenated and aligned with MAFFT v7. A bacterial phylogeny was then built with PhyML v3.0 using the GTR GAMMA substitution model with 1,000 bootstrap replicates.

### Data Visualization and Statistical Analysis

The visualization of phylogenies and their related information (host taxonomy, bacterial strain, *Wolbachia* supergroup, protein domains) was done using the online tool iTOL v4.3.3 (Letunic and Bork 2007, https://itol.embl.de/; last accessed August 20, 2020). All statistical analyses were performed in the R software (R Core Team 2013). The congruence between the phylogenies of *cifA* and *cifB* homologs as well as their concatenation, and their respective *Wolbachia* strains were tested using Mantel tests on the pairwise patristic distances between sequences (1,000 permutations). Additionally, ParaFit with 9,999 permutations implemented in CopyCat v2.0 (Meier-Kolthoff et al. 2007) was used to examine cophylogenetic signals between trees and visualize the contribution of individual links between them. Finally, we compared the observed mean phylogenetic distances between *cif* gene pairs occurring within the same genome to a random distribution of mean distances generated by randomly permuting *cif* genes between genomes (1,000 permutations).

## Supplementary Material

Supplementary data are available at *Molecular Biology and Evolution* online.

## Supplementary Material

msaa209_supplementary_data
