# Supplementary material for: Life and Death of Selfish Genes: Comparative Genomics Reveals the Dynamic Evolution of Cytoplasmic Incompatibility
Source: Mol Biol Evol. 2020 Aug 14;38(1):2–15. doi: 10.1093/molbev/msaa209 (PMC7783169; doi:10.1093/molbev/msaa209)
Supplement: msaa209_supplementary_data [file msaa209_supplementary_data.zip › Table S1.pdf]

Wolbachia strain	*Wolbachia* supergroup	Host taxonomy	Native host	Accession number	Phenotype
-	A	Coleoptera	*Diabrotica virgifera virgifera 1*	SAMN02373824[c]	CI[a] [1]
wAna	A	Diptera	*Drosophila ananassae*	AAGB00000000.1	CI[a,b] [2,3]
wAra	A	Diptera	*Drosophila arawakana*	JAATKZ000000000	CI[b] [2]
wAu	A	Diptera	*Drosophila simulans*	LK055284.1	no reproductive phenotype[a,b] [4,5]
wBai	A	Diptera	*Drosophila baimaii*	JAATLA000000000	unknown
wBic	A	Diptera	*Drosophila bicornuta*	JAATLB000000000	unknown
wBif	A	Diptera	*Drosophila bifasciata*	JAATLC000000000	MK[a] [6]
wBor	A	Diptera	*Drosophila borealis*	JAATLD000000000	MK[a] [7]
wHa	A	Diptera	*Drosophila simulans*	CP003884.1	CI[a] [8]
wInc_CU	A	Diptera	*Drosophila incompta*	CP011148.1	unknown
wInc_SM	A	Diptera	*Drosophila incompta*	CP011149.1	unknown
wMel	A	Diptera	*Drosophila melanogaster*	NC_002978.6	CI[a,b] [9,10]
wMelPop	A	Diptera	*Drosophila melanogaster*	NZ_AQQE00000000.1	CI[a,b] [11,12]
wNeo	A	Diptera	*Drosophila neotestacea*	JAATLE000000000	None[a] [13]
wNik	A	Diptera	*Drosophila nikananu*	CP050530	unknown
wOrie	A	Diptera	*Drosophila orientacea*	JAATLF000000000	unknown
wRec	A	Diptera	*Drosophila recens*	NZ_JQAM00000000.1	CI[a] + MK[b] [14]
wRi	A	Diptera	*Drosophila simulans*	NC_012416.1	CI[a] [15]
wSpc	A	Diptera	*Drosophila subpulchrella*	NZ_NTHL00000000.1	no reproductive phenotype[a] [16]
wStv	A	Diptera	*Drosophila sturtevanti*	CP050531	CI[b] [2]
wSuz	A	Diptera	*Drosophila suzukii*	NZ_CAOU00000000.2	no phenotype to weak CI[a,b] [16,17]
wTri-2	A	Diptera	*Drosophila triauraria*	JAATLG000000000	CI[a,b] [2,18]
wTro	A	Diptera	*Drosophila tropicalis*	JAATLH000000000	no reproductive phenotype[b] [2]
wWil	A	Diptera	*Drosophila willistoni*	AAQP00000000.1	unknown
wYak	A	Diptera	*Drosophila yakuba 1*	SAMN04044077[c]	no phenotype to weak CI[a,b] [2,19–21]
wDacA	A	Hemiptera	*Dactylopius coccus*	NZ_LSYX00000000.1	unknown
-	A	Hymenoptera	*Acromyrmex echinatior*	SAMEA762107[c]	unknown
-	A	Hymenoptera	*Biorhiza pallida 1*	SAMEA2053316[c]	unknown
-	A	Hymenoptera	*Ceratina calcarata 1*	SAMN04210145[c]	unknown
-	A	Hymenoptera	*Cynipini 1*	SAMEA1965365[c]	unknown
-	A	Hymenoptera	*Diachasma alloeum*	SAMN03701895[c]	unknown
-	A	Hymenoptera	*Pediaspis aceris 2*	SAMEA3925673[c]	unknown
-	A	Hymenoptera	*Pseudomyrmex sp PSW 54*	SAMN03275520[c]	unknown
wNfe	A	Hymenoptera	*Nomada ferruginata*	NZ_LYUY00000000.1	unknown
wNfla	A	Hymenoptera	*Nomada flava*	NZ_LYUW00000000.1	unknown
wNleu	A	Hymenoptera	*Nomada leucophthalma*	NZ_LYUV00000000.1	unknown
wNpa	A	Hymenoptera	*Nomada panzeri*	NZ_LYUX00000000.1	unknown
wUni	A	Hymenoptera	*Muscidifurax uniraptor*	NZ_ACFP00000000.1	P[a] [22]
wVitA	A	Hymenoptera	*Nasonia vitripennis*	NZ_MUJM00000000.1	CI[a] [23]
-	B	Coleoptera	*Bembidion lapponicum*	SAMN04276907[c]	unknown
-	B	Coleoptera	*Diploeciton nevermanni*	SAMN05860871[c]	unknown
-	B	Coleoptera	*Ecitophya simulans*	SAMN05833357[c]	unknown
-	B	Diptera	*Anopheles species A*[d]	SAMEA3911293[c]	unknown
wAlbB	B	Diptera	*Aedes albopictus*	NZ_CAGB00000000.1	CI[a,b] [5,24]
wNo	B	Diptera	*Drosophila simulans*	NC_021084.1	CI[a] [8]
wPipJHB	B	Diptera	*Culex pipiens* complex	ABZA00000000.1	unknown
wPipMol	B	Diptera	*Culex pipiens* complex	NZ_CACK00000000.1	CI[a] [25]

wPipPel	B	Diptera	*Culex pipiens* complex	AM999887.1	CI[a,b] [26]
-	B	Hemiptera	*Gerris buenoi 1*	SAMN02439785[c]	unknown
-	B	Hemiptera	*Maconellicoccus hirsutus*	SAMEA3699093[c]	unknown
-	B	Hemiptera	*Mycopsylla fici 1*	SAMN04226368[c]	unknown
WBT1	B	Hemiptera	*Bemisia tabaci*	NZ_FMKF00000000.1	unknown
wDacB	B	Hemiptera	*Dactylopius coccus*	NZ_LSYY00000000.1	unknown
wDi	B	Hemiptera	*Diaphorina citri*	NZ_AMZJ00000000.1	unknown
wStri	B	Hemiptera	*Laodelphax striatella*	NZ_LRUH00000000.1	CI[a] [27]
-	B	Hymenoptera	*Diplolepis spinosa 1*	SAMEA3930570[c]	unknown
-	B	Hymenoptera	*Isocolus centaureae 1*	SAMEA3930555[c]	unknown
wTpre	B	Hymenoptera	*Trichogramma pretiosum*	NZ_CM003641.1	P[a] [28]
wVitB	B	Hymenoptera	*Nasonia vitripennis*	AERW00000000.1	CI[a] [23]
-	B	Lepidoptera	*Delias oraia*	SAMEA3911293[c]	unknown
-	B	Lepidoptera	*Operophtera brumata*	SAMN03121611[c]	unknown
-	B	Lepidoptera	*Pararge aegeria*	SAMN02688782[c]	unknown
-	B	Lepidoptera	*Polygonia c-album*	SAMN02688783[c]	unknown
wAus	B	Lepidoptera	*Plutella Australiana*	MRWX00000000.1	unknown
wBol1	B	Lepidoptera	*Hypolimnas bolina*	CAOH01000001	MK[a] + CI[a] [29]
wOo	C	Nematode	*Onchocerca ochengi*	NC_018267.1	M
wOvc	C	Nematode	*Onchocerca volvulus*	NZ_HG810405.1	M
wBm	D	Nematode	*Brugia malayi*	NC_006833.1	M
wFol	E	Collembola	*Folsomia candida*	NZ_CP015510.2	putatively P[a] [30]
wCle	F	Hemiptera	*Cimex lectularius*	NZ_AP013028.1	M [31]
wPpe	L	Nematode	*Pratylenchus penetrans*	NZ_MJMG00000000.1	M

CI: Cytoplasmic incompatibility
MK: Male-killing
P: Parthenogenesis
M: Mutualism / obligate symbiosis
[a]phenotype in original host species
[b]phenotype upon artificial transfer into a different host species
[c]arthropod sequencing data used to assemble *Wolbachia* genomes in [32]
[d]annotated as Anopheles gambiae but see [33]

References

1. Giordano R, Jackson JJ, Robertson HM. The role of Wolbachia bacteria in reproductive incompatibilities and hybrid zones of Diabrotica beetles and Gryllus crickets. Proc Natl Acad Sci. 1997;94: 11439–11444.
2. Martinez J, Ok S, Smith S, Snoeck K, Day JP, Jiggins FM. Should Symbionts Be Nice or Selfish? Antiviral Effects of Wolbachia Are Costly but Reproductive Parasitism Is Not. PLOS Pathog. 2015;11: e1005021.
3. Bourtzis K, Nirgianaki A, Markakis G, Savakis C. Wolbachia infection and cytoplasmic incompatibility in Drosophila species. Genetics. 1996;144: 1063–1073.
4. Hoffmann A, Clancy D, Duncan J. Naturally-occurring Wolbachia infection in Drosophila simulans that does not cause cytoplasmic incompatibility. Heredity. 1996;76: 1–8.
5. Ant TH, Herd CS, Geoghegan V, Hoffmann AA, Sinkins SP. The Wolbachia strain wAu provides highly efficient virus transmission blocking in Aedes aegypti. PLOS Pathog. 2018;14: e1006815.
6. Hurst GD, Johnson AP, Schulenburg JH, Fuyama Y. Male-killing Wolbachia in Drosophila: a temperature-sensitive trait with a threshold bacterial density. Genetics. 2000;156: 699–709.
7. Sheeley SL, McAllister BF. Mobile male-killer: similar Wolbachia strains kill males of divergent Drosophila hosts. Heredity. 2009;102: 286–92. doi:10.1038/hdy.2008.126
8. Merçot H, Llorente B, Jacques M, Atlan A, Montchamp-Moreau C. Variability within the Seychelles cytoplasmic incompatibility system in Drosophila simulans. Genetics. 1995;141: 1015–1023.
9. Blagrove MSC, Arias-Goeta C, Failloux A-B, Sinkins SP. Wolbachia strain wMel induces cytoplasmic incompatibility

and blocks dengue transmission in Aedes albopictus. Proc Natl Acad Sci U S A. 2012;109: 255–60.

10. Poinsot D, Bourtzis K, Markakis G, Savakis C. Wolbachia Transfer from Drosophila melanogaster into D. simulans: Host Effect and Cytoplasmic Incompatibility Relationships. Genetics. 1998;150: 227–237.

11. McMeniman C, Lane R, Cass B, Fong A, Sidhu M, Wang Y, et al. Stable Introduction of a Life-Shortening Wolbachia Infection into the Mosquito Aedes aegypti. Science. 2009;323: 141–144.

12. McMeniman CJ, Lane AM, Fong AWC, Voronin D a, Iturbe-Ormaetxe I, Yamada R, et al. Host adaptation of a Wolbachia strain after long-term serial passage in mosquito cell lines. Appl Environ Microbiol. 2008;74: 6963–9.

13. Jaenike J, Stahlhut JK, Boelio LM, Unckless RL. Association between Wolbachia and Spiroplasma within Drosophila neotestacea: an emerging symbiotic mutualism? Mol Ecol. 2010;19: 414–25.

14. Jaenike J. Spontaneous emergence of a new Wolbachia phenotype. Evolution. 2007;61: 2244–2252.

15. Hoffmann A, Turelli M, Simmons G. Unidirectional incompatibility between populations of Drosophila simulans. Evolution. 1986;40: 692–701.

16. Hamm CA, Begun DJ, Vo A, Smith CCR, Saelao P, Shaver AO, et al. Wolbachia do not live by reproductive manipulation alone: infection polymorphism in Drosophila suzukii and D. subpulchrella. Mol Ecol. 2014;23: 4871–4885.

17. Cattel J, Nikolouli K, Andrieux T, Martinez J, Jiggins F, Charlat S, et al. Back and forth Wolbachia transfers reveal efficient strains to control spotted wing drosophila populations. J Appl Ecol. 2018;55: 2408–2418.

18. Turelli M, Cooper BS, Richardson KM, Chiu JC, Conner WR, Hoffmann AA, et al. Rapid Global Spread of w Ri-like Wolbachia across Multiple Drosophila Report Rapid Global Spread of wRi-like Wolbachia across Multiple Drosophila. Curr Biol. 2018;28: 963–971.

19. Zabalou S, Apostolaki A, Pattas S, Veneti Z, Paraskevopoulos C, Livadaras I, et al. Multiple rescue factors within a Wolbachia strain. Genetics. 2008;178: 2145–60.

20. Zabalou S, Charlat S, Nirgianaki A, Lachaise D, Merçot H, Bourtzis K. Natural Wolbachia infections in the Drosophila yakuba species complex do not induce cytoplasmic incompatibility but fully rescue the wRi modification. Genetics. 2004;167: 827–34.

21. Cooper BS, Ginsberg PS, Turelli M, Matute DR. Wolbachia in the Drosophila yakuba complex: Pervasive frequency variation and weak cytoplasmic incompatibility, but no apparent effect on reproductive isolation. Genetics. 2017;205: 333–351.

22. Stouthamer R, Breeuwer JAJ, Luck RF, Werren JH. Molecular identification of microorganisms associated with parthenogenesis. Nature. 1993;361: 66–68.

23. Bordenstein SR, Werren JH. Effects of A and B Wolbachia and host genotype on interspecies cytoplasmic incompatibility in Nasonia. Genetics. 1998;148: 1833–1844.

24. Xi Z, Dean JL, Khoo C, Dobson SL. Generation of a novel Wolbachia infection in Aedes albopictus (Asian tiger mosquito) via embryonic microinjection. Insect Biochem Mol Biol. 2005;35: 903–910.

25. Walker T, Song S, Sinkins SP. Wolbachia in the Culex pipiens Group Mosquitoes: Introgression and Superinfection. J Hered. 2008;100: 192–196.

26. Sinkins SP, Walker T, Lynd AR, Steven AR, Makepeace BL, Godfray HCJ, et al. Wolbachia variability and host effects on crossing type in Culex mosquitoes. Nature. 2005;436: 257–260.

27. Noda H, Koizumi Y, Zhang Q, Deng K. Infection density of Wolbachia and incompatibility level in two planthopper species, Laodelphax striatellus and Sogatella furcifera. Insect Biochem Mol Biol. 2001;31: 727–737.

28. Stouthamer R, Kazmer DJ. Cytogenetics of microbe-associated parthenogenesis and its consequences for gene flow in Trichogramma wasps. Heredity. 1994;73: 317–327.

29. Hornett EA, Duplouy AMR, Davies N, Roderick GK, Wedell N, Hurst GDD, et al. You can't keep a good parasite down: evolution of a male-killer suppressor uncovers cytoplasmic incompatibility. Evolution. 2008;62: 1258–1263.

30. Frati F, Negri I, Fanciulli PP, Pellecchia M, De Paola V, Scali V, et al. High levels of genetic differentiation between Wolbachia-infected and non-infected populations of Folsomia candida (Collembola, Isotomidae). Pedobiologia (Jena). 2004;48: 461–468.

31. Hosokawa T, Koga R, Kikuchi Y, Meng X-Y, Fukatsu T. Wolbachia as a bacteriocyte-associated nutritional mutualist. Proc Natl Acad Sci. 2010;107: 769–774.

32. Pascar J, Chandler CH. A bioinformatics approach to identifying *Wolbachia* infections in arthropods. PeerJ. 2018;6: e5486.

33. Chrostek E, Gerth M. Is Anopheles gambiae a Natural Host of Wolbachia? MBio. 2019;10: e00784-19.
