# Supplementary figures and images for: Life and Death of Selfish Genes: Comparative Genomics Reveals the Dynamic Evolution of Cytoplasmic Incompatibility
Source: Mol Biol Evol. 2020 Aug 14;38(1):2–15. doi: 10.1093/molbev/msaa209 (PMC7783169; doi:10.1093/molbev/msaa209)

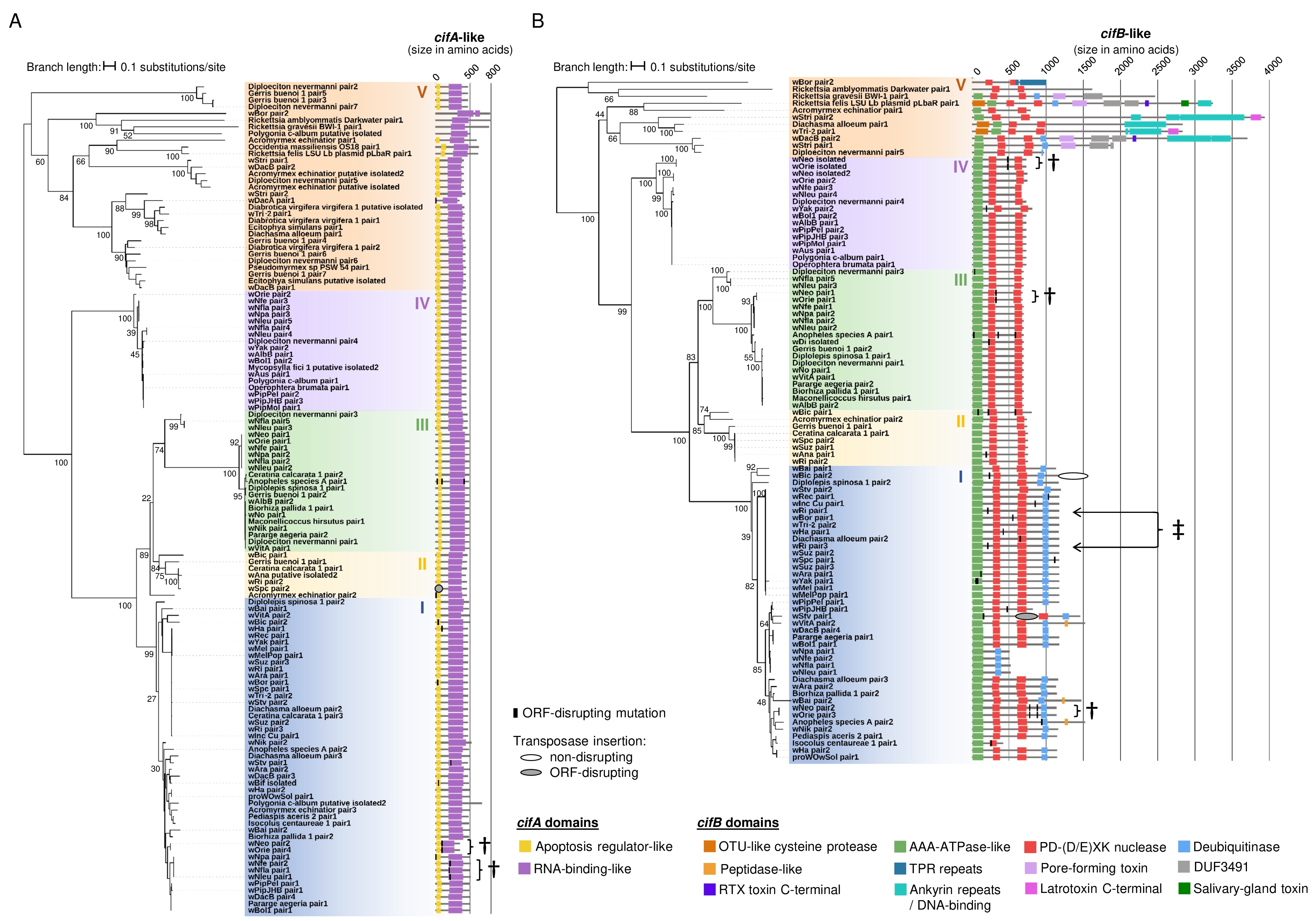

Supplement: msaa209_supplementary_data [file msaa209_supplementary_data.zip › Figure S1.jpg]

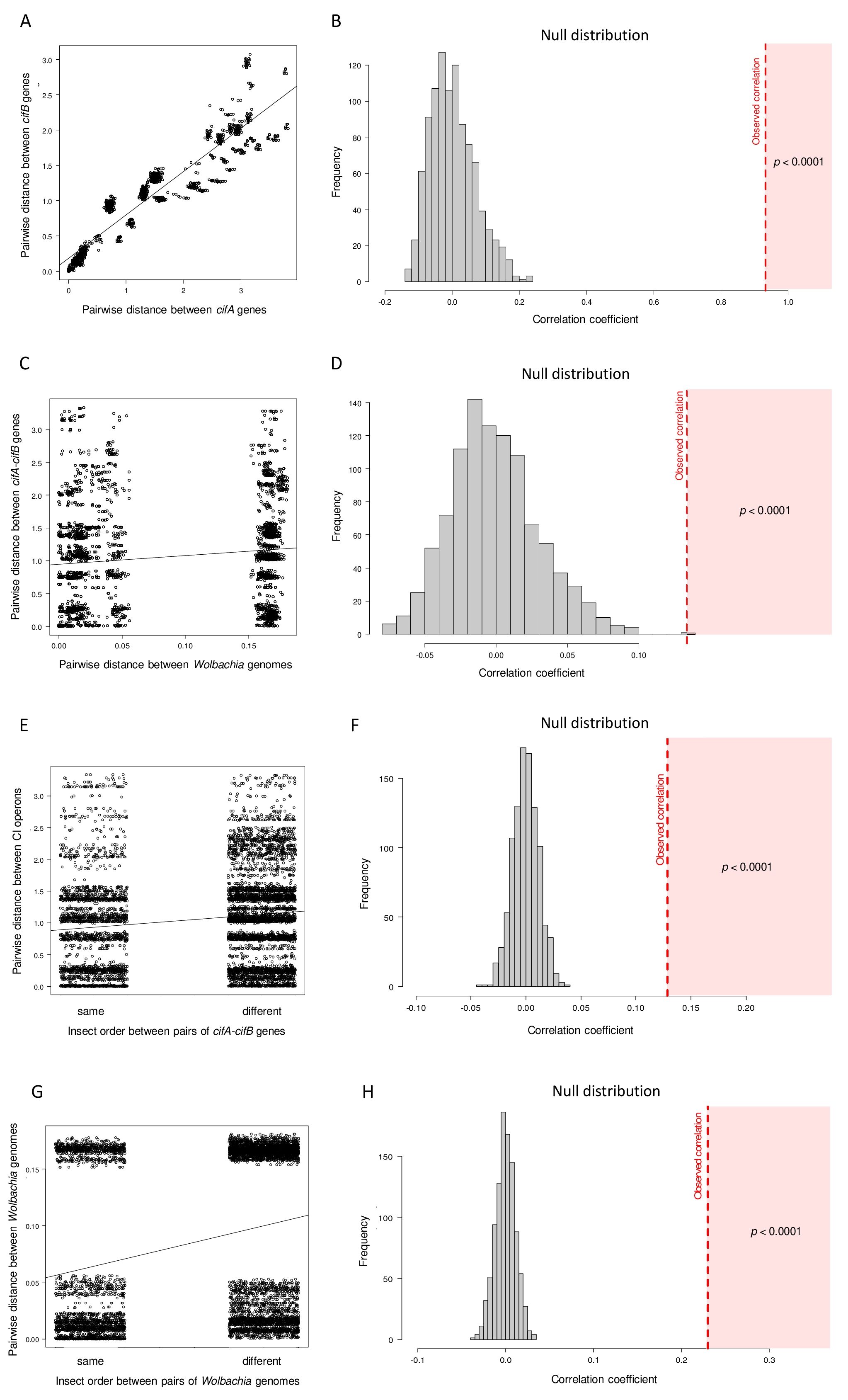

Supplement: msaa209_supplementary_data [file msaa209_supplementary_data.zip › Figure S2.jpg]

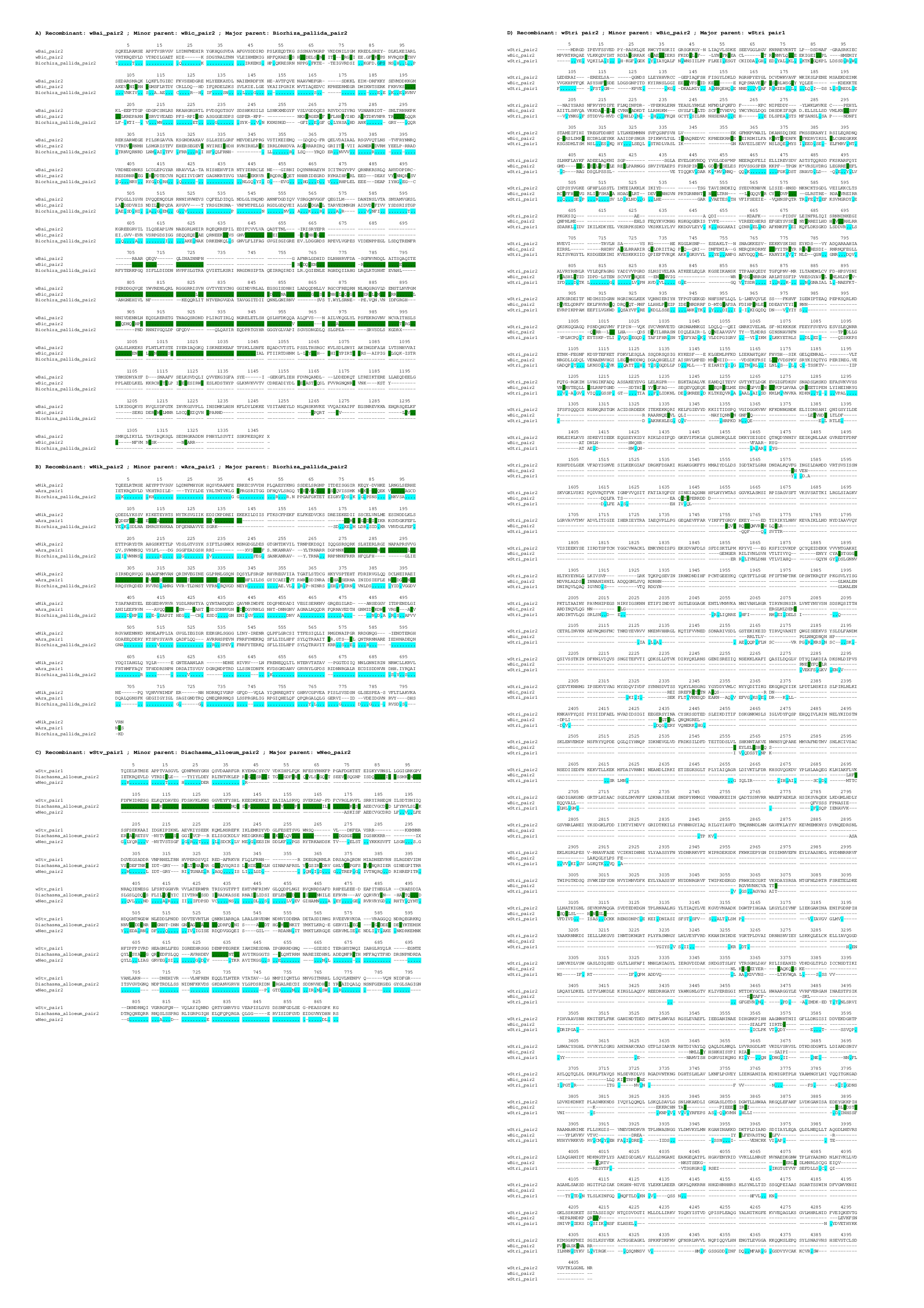

Supplement: msaa209_supplementary_data [file msaa209_supplementary_data.zip › Figure S3.jpg]

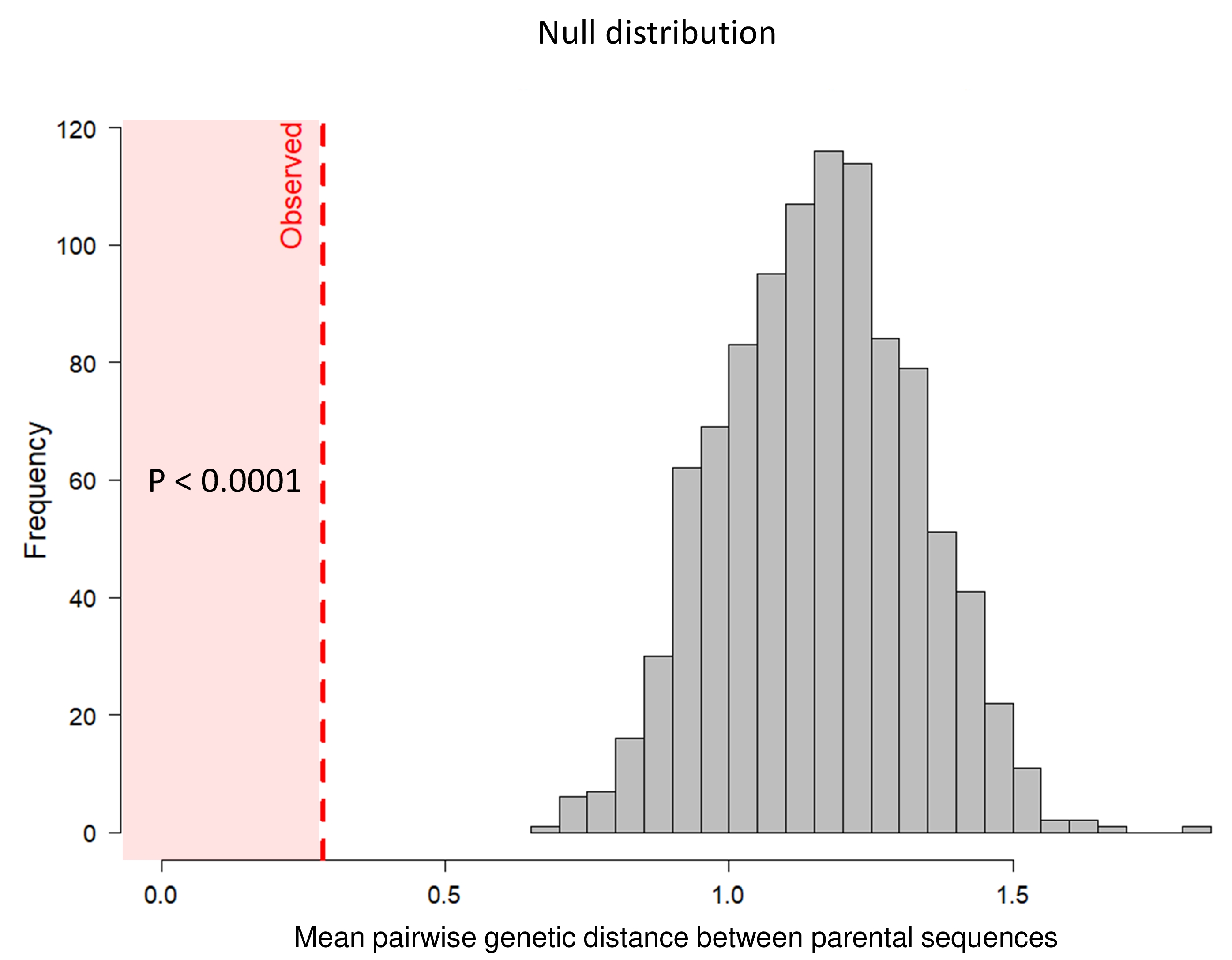

Supplement: msaa209_supplementary_data [file msaa209_supplementary_data.zip › Figure S4.jpg]

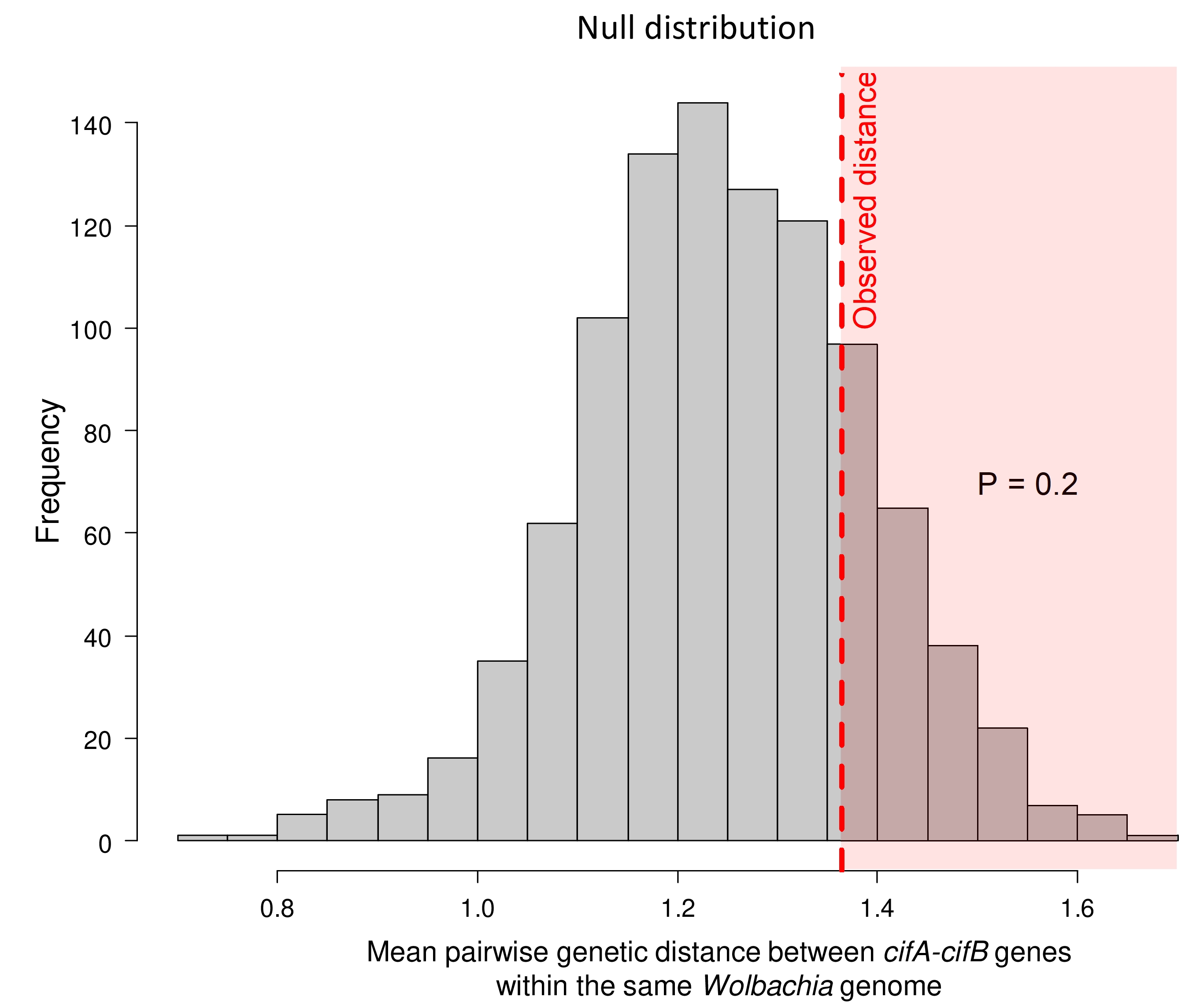

Supplement: msaa209_supplementary_data [file msaa209_supplementary_data.zip › Figure S5.jpg]

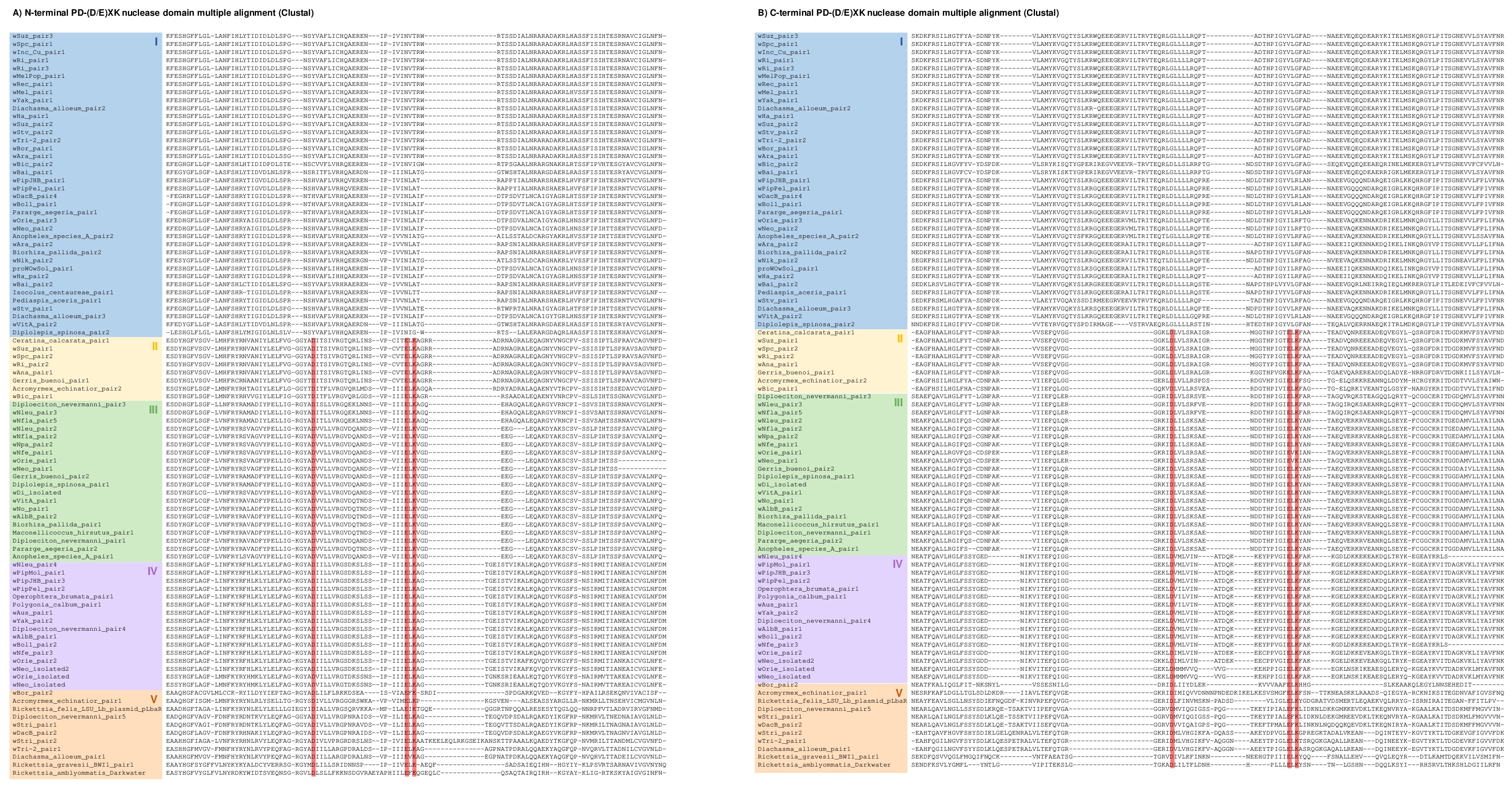

Supplement: msaa209_supplementary_data [file msaa209_supplementary_data.zip › Figure S6.jpg]

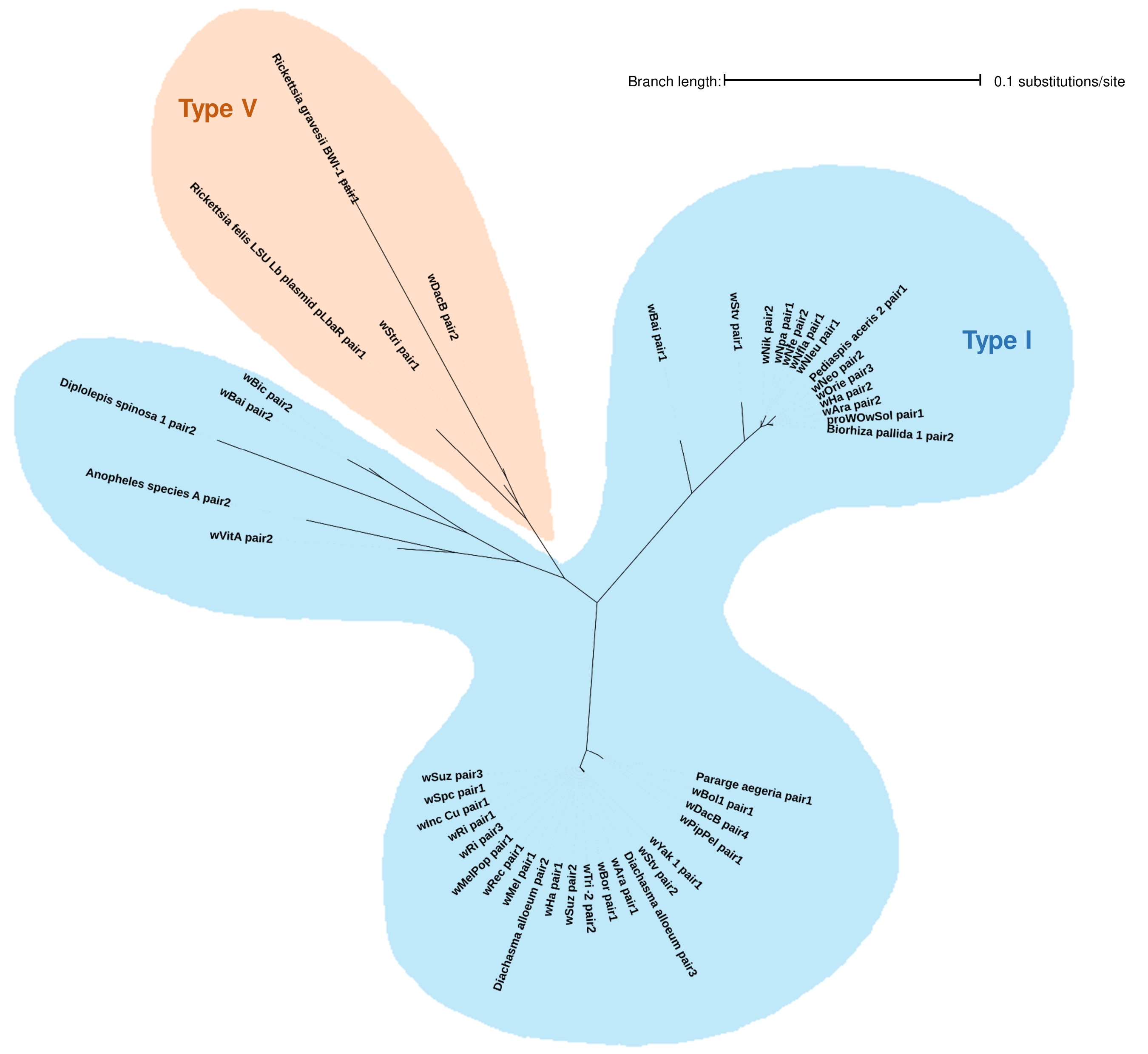

Supplement: msaa209_supplementary_data [file msaa209_supplementary_data.zip › Figure S7.jpg]
